# Harnessing axonal transport to map reward circuitry: Differing brain-wide projections from medial prefrontal cortical domains

**DOI:** 10.3389/fcell.2023.1278831

**Published:** 2023-11-30

**Authors:** Elaine L. Bearer, Christopher S. Medina, Taylor W. Uselman, Russell E. Jacobs

**Affiliations:** ^1^ Department of Pathology, University of New Mexico Health Sciences Center, Albuquerque, NM, United States; ^2^ Division of Biology and Biological Engineering, California Institute of Technology, Pasadena, CA, United States; ^3^ Zilkha Neurogenetic Institute, USC Keck School of Medicine, Los Angeles, CA, United States

**Keywords:** axonal transport, medial prefrontal cortex (mPFC), manganese-enhanced magnetic resonance imaging (MEMRI), calcium analog, statistical parametric mapping (SPM), anterior cingulate area (ACA) and infralimbic-prelimbic cortex (IL/PL), amygdala, hypthalamus

## Abstract

Neurons project long axons that contact other distant neurons. Neurons in the medial prefrontal cortex project into the limbic system to regulate responses to reward or threat. Diminished neural activity in prefrontal cortex is associated with loss of executive function leading to drug use, yet the specific circuitry that mediate these effects is unknown. Different regions within the medial prefrontal cortex may project to differing limbic system nuclei. Here, we exploited the cell biology of intracellular membrane trafficking, fast axonal transport, to map projections from two adjacent medial prefrontal cortical regions. We used Mn(II), a calcium analog, to trace medial prefrontal cortical projections in the living animal by magnetic resonance imaging (MRI). Mn(II), a contrast agent for MRI, enters neurons through voltage-activated calcium channels and relies on kinesin-1 and amyloid-precursor protein to transport out axons to distal destinations. Aqueous MnCl_2_ together with fluorescent dextran (3–-5 nL) was stereotactically injected precisely into two adjacent regions of the medial prefrontal cortex: anterior cingulate area (ACA) or infralimbic/prelimbic (IL/PL) region. Projections were traced, first live by manganese-enhanced MRI (MEMRI) at four time points in 3D, and then after fixation by microscopy. Data-driven unbiased voxel-wise statistical maps of aligned normalized MR images after either ACA or IL/PL injections revealed statistically significant progression of Mn(II) over time into deeper brain regions: dorsal striatum, globus pallidus, amygdala, hypothalamus, substantia nigra, dorsal raphe and locus coeruleus. Quantitative comparisons of these distal accumulations at 24 h revealed dramatic differences between ACA and IL/PL injection groups throughout the limbic system, and most particularly in subdomains of the hypothalamus. ACA projections targeted dorsomedial nucleus of the hypothalamus, posterior part of the periventricular region and mammillary body nuclei as well as periaqueductal gray, while IL/PL projections accumulated in anterior hypothalamic areas and lateral hypothalamic nuclei as well as amygdala. As hypothalamic subsegments relay CNS activity to the body, our results suggest new concepts about mind-body relationships and specific roles of distinct yet adjacent medial prefrontal cortical segments. Our MR imaging strategy, when applied to follow other cell biological processes in the living organism, will undoubtedly lead to an expanded perspective on how minute details of cellular processes influence whole body health and wellbeing.

## Graphical Abstract

**Figure:**
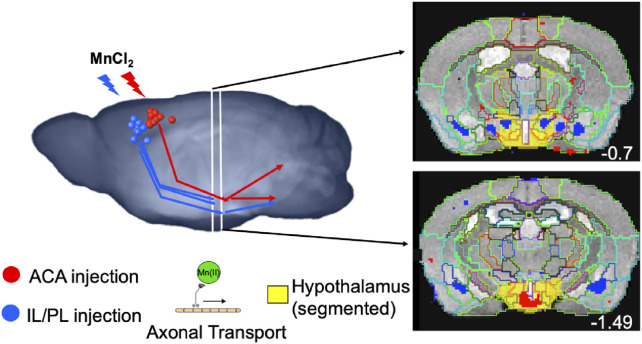
Tract tracing after MnCl_2_ stereotactic intracerebral injection is performed by time-lapse MRI in living mice, based on harnessing the intracellular axonal transport machinery, kinesin and microtubules. Volumes of statistically significant, Mn(II)-dependent, image enhancements report on distal accumulations brain wide that can be measured segment-wise for statistical comparisons of the relative strength of the projection contacts. Targets of neuronal projections from the medial prefrontal cortex differ between anterior cingulate (ACA, red) and infralimbic-prelimbic (IL/PL, blue) areas. While neurons in ACA project to central amygdala, posterior hypothalamic regions, periaqueductal gray, dorsal raphe, and locus coeruleus; those in IL/PL project to basolateral amygdala, anterior hypothalamic subsegments, central superior raphe and pons.

## Introduction

Medial prefrontal pyramidal neurons project long axons deep into distant brain regions. These projections have been mapped by experimentally hijacking endogenous membrane trafficking machinery, axonal transport, that feeds the neuronal synapse by introducing labeled tracers into that transport system, either distally for retrograde transport in the rat (Gabbott et al., 2005) or within the forebrain for anterograde transport in mice (Fillinger et al., 2018). A variety of clever molecular methods to introduce labeled tracers into the specific subgroups of neurons have been developed to map projections from the cortex (Zingg et al., 2014) and other sites. The mouse brain connectivity project at the Allen Institute for Brain Science has mapped projections using fluorescence microscopy in individual mouse brains after fixation, including those from the medial prefrontal cortex (mPFC) (https://connectivity.brain-map. org/projection) (Oh et al., 2014) which may add detail to the tractography of prefrontal projections by diffusion tensor magnetic resonance imaging (MRI) (Aydogan et al., 2018). While very exciting, the optogenetic approach only reveals physiological connections activated experimentally along traced pathways to candidate regions, such as those from the forebrain to nucleus accumbens and basolateral amygdala (Riga et al., 2014), but not their normal activity or brain-wide influence. Only with brain-wide imaging can the full picture of projections be mapped (Shi et al., 2022). While there is a long history of results obtained from the histologic tract traced by microscopy, this approach is somewhat limited by the opacity of the brain, which necessitates euthanasia of animals for subsequent optical imaging, either by sectioning (Sesack and Pickel, 1992; Gabbott et al., 2005; Fillinger et al., 2018), by serial sectioning (Shi et al., 2022) or by rendering the brain transparent for whole brain light-sheet microscopy (Jing et al., 2018). Histologic tracers are typically large molecules labeled either enzymatically with an antigen or with a fluorescent tag. Whether injected or expressed from an exogenous vector, these tracers typically take longer (weeks) to accumulate distally in sufficient amounts for detection by microscopy. Furthermore, it can be challenging to identify target regions, and often data from only one individual are reported, the injection sites are varied, the injection volume is large, and consistent patterns, statistically significant, between many individuals are difficult to obtain. Each type of tracing provides a differing perspective on the circuitry.

Tracing projections is critical to our understanding of brain circuitry. To witness functional connections in living animals, we developed a tracer detectable by MRI, Mn(II) (Bearer et al., 2007a; Bearer et al., 2007b; Uselman et al., 2022). Initially proposed as an MR contrast agent for axonal tracing by Koretsky’s group (Pautler and Koretsky, 2002), Mn(II) provides a bright signal in T_1_-weighted MRI, enters cells through voltage-gated calcium channels (Narita et al., 1990; Bedenk et al., 2018) and thus is a calcium indicator. Mn(II) is trafficked in axons by the kinesin-based microtubule vesicular anterograde transport (Pautler, 2006; Bearer et al., 2007a; Medina et al., 2017a), crosses active synapses (Pautler et al., 2003; Pautler, 2006; Bearer et al., 2007a), and tracing is performed in living animals.

Automated computational preprocessing of resultant MR images permits the rapid analysis of large numbers of animals sufficient for statistical power. Projection mapping by manganese-enhanced MRI (MEMRI) informs on functional connections between neurons, while tractography by diffusion-weighted magnetic resonance imaging informs on the anatomy of axon bundles. Maps of axonal projections emanating from a localized intracerebral injection of MnCl_2_ are acquired by MEMRI. MEMRI has traced projections from nares to the olfactory bulb (Pautler and Koretsky, 2002), from the retina to the visual cortex (Watanabe et al., 2002), from the amygdala to the hippocampus (Pautler et al., 2003), from CA3 of the hippocampus to the septal nuclei in the basal forebrain (Bearer et al., 2007b; Gallagher et al., 2012; Medina et al., 2017a; Bearer et al., 2018; Medina et al., 2019), and from prefrontal cortex globally into many regions throughout the deeper part of the brain (Bearer et al., 2009a; Zhang et al., 2010; Gallagher et al., 2013). We used MEMRI to define differences in axonal transport rates from hippocampus to basal forebrain in aging and in specific disease states, from Down syndrome to Alzheimer’s disease (Bearer et al., 2007b; Gallagher et al., 2012; Gallagher et al., 2013; Medina et al., 2017a; Bearer et al., 2018; Medina et al., 2019). Thus, MEMRI could be useful for the studies of cognition, memory, and learning. It is to be noted that manganese tract-tracing is particularly useful for diffuse projections not otherwise amenable to diffusion tensor tractography, where the distal accumulations of Mn(II) can be detected and mapped and intensity values measured as a proxyfor connections and for the relative rates of transport to distal destinations.

Phineas Gage’s personality change after his misadventure in the 1840s when the iron rod plunged through his forebrain attracted attention to this region of the brain in decision making, executive functions, and emotions (Damasio et al., 1994). Since then, much progress has been made discovering the functional correlates of that region, its projections into the brain, and the reciprocal effects of substance use (Volkow et al., 1996; Frankin et al., 2002). The nomenclature and segmentation of the medial prefrontal cortex has been under review, with some proposals for a vocabulary that aligns with functional data from other species (van Heukelum et al., 2020). Our nomenclature is based on the Allen Institute Mouse Brain Reference Atlas (Lein et al., 2007) with some correlations to the Paxinos atlas and its more recent update (Paxinos and Franklin, 2001; Vogt and Paxinos, 2014). Our work with MEMRI tract-tracing demonstrated that the disruption of the three molecular targets of cocaine, SERT, DAT, and NET, altered the functional anatomy of medial prefrontal cortical projections brain-wide (Bearer et al., 2009a; Bearer et al., 2009b; Zhang et al., 2010; Gallagher et al., 2013). Phineas Gage’s injury encompassed a large area in the prefrontal cortex that has been subdivided in more detail by recent experiments. The anterior cingulate and infralimbic/prelimbic areas within the larger medial prefrontal cortex may project to different deeper regions in the brainstem, limbic, and neuromodulatory structures (Gabbott et al., 2005; Fillinger et al., 2018). These functional connections are not fully realized yet.

Here, we consider a meta-dimensional approach to map connections from the two different regions of the medial forebrain, as detected by Mn(II) accumulations. Our approach differs from previous studies by allowing us to collect and compare data from the two sets of animals with each set injected in a different location. After stereotactic intracerebral injections, we collected MRI brain-wide in 4D at 100 μm^3^ resolution, the level of a few neurons. Our sample size was large enough to attain statistical significance in unbiased statistical analyses performed on images from each cohort. As the C57BL/6 congenic mouse has minimal inter-individual anatomical differences, this approach produced a map of the average projection anatomy common to all individuals in each cohort. Our innovative quantification of fractional accumulation volumes using our new annotated *InVivo* Atlas provides a new way to measure the relative strength of projections to distal locations. By tracing these forebrain projections brain-wide in a living experimental model over time, aspects of controversies regarding species differences in prefrontal cortical projections may be explored, and differences and similarities between projections from these two cortical brain regions, which are defined by their position and anatomy, were revealed. Using an anatomical atlas as pivot to compare between species (Barron et al., 2021), our results amy also inform on the human condition.

Using a pre-injection MR image to program a computer-driven stereotactic injection apparatus, we acquired tightly focused intracerebral injection sites, each reproducible across a dozen individuals. Because Mn(II) provides a bright signal, the volume needed for imaging is very small (three to 5 nL) (Bearer et al., 2022) and produces no detectable permanent damage by histopathology or electrophysiology at this volume and dosage (Uselman et al., 2022). Mn(II) travels quickly, at 5 mm/h in the optic nerve (Bearer et al., 2007a), similar to vesicular transport in the rat optic nerve (Elluru et al., 1995). Thus, MR images captured successively at 6 h–24 h reported on arrival *via* transport at distal destinations in living animals (Bearer et al., 2009a; Zhang et al., 2010; Gallagher et al., 2012; Gallagher et al., 2013; Medina et al., 2017a; Medina et al., 2019). By 24 h, the Mn(II) signal is detected as far as 1.5 cm from the injection site, whereas for the histologic tracer, rhodamine dextran, 3 weeks is necessary for detection at much shorter distances and requires sacrifice and fixation to visualize (Bearer et al., 2009a; Zhang et al., 2010; Gallagher et al., 2013). Because the brain is transparent to MRI, the dynamics of transport in living animals can be followed. As we have shown, Mn(II) transport is in part dependent on the microtubule-based molecular motor, kinesin-1, and on the transmembrane protein, amyloid precursor protein, an adaptor for kinesin-1 (Satpute-Krishnan et al., 2006; Gallagher et al., 2012; Bearer and Wu, 2019). Thus, MEMRI reports on global connections in living mice at relatively short time intervals. Electrical activity does not affect the rate of Mn(II) axonal transport within axons (Bearer et al., 2007a). However, Mn(II) preferentially crosses active synapses, accumulating in post-synaptic neurons (Bearer et al., 2007a). Thus, MEMRI track-tracing reveals active trans-synaptic projections, but not physical connections of silent neurons.

The novelty of this work goes well beyond what has already been reported in several ways. First, MEMRI tract-tracing differs from histological approaches as, together with our computational imaging processing pipeline, we obtain statistically significant information brain-wide in the living, freely moving animal, which is not possible with histologic tracing. Second, while we have previously reported MEMRI track-tracing from medial prefrontal cortex (Bearer et al., 2009b; Zhang et al., 2010; Gallagher et al., 2013), here we further expand on this imaging technology with computational tools that obtain quantitative information about projection strength and fractional accumulation volumes. This new method deploys automated segmentation with our new annotated digital atlas to extract volumetric measurements of Mn(II) accumulations at each segment receiving input across progressive time points after injection. No other technology or analysis can yield this critical information. This innovation allowed us for the first time to measure and compare levels of projection activity at distal destinations throughout the brain. Moreover, our annotated digital atlas identifies the specific locations of distal projection anatomy more precisely than has been possible with visual comparisons between the microscopy of tracers and histologic atlases, as has been the method of choice for histologic tract-tracing previously. Finally, statistical analysis, made possible with our computational ability to aggregate a sufficient sample size of images for statistical power, allowed us for the first time to map and measure the average, consistent, basic projection map common among individuals from two different injection sites.

Computational processing of whole brain image stacks allows the sophisticated statistical analyses of large datasets containing hundreds of images from many living individuals, each captured successively at multiple time points. Initially, the brain image is segmented from the living animal’s whole head image by masking non-brain tissues (Delora et al., 2016); then, all images are normalized, both spatially and intensity-wise, and finally anatomically aligned into a uniform 3D matrix (Medina et al., 2017b). Aligned datasets are subjected to unbiased, voxel-wise statistical parametric mapping or region of interest intensity measurements to map and measure projection patterns and connectivity (Bearer et al., 2009a; Zhang et al., 2010; Gallagher et al., 2013; Uselman et al., 2020). Here, we compare distal projections from injections into two distinct adjacent regions in the medial prefrontal cortex. First, we explore our new dataset obtained after injections into the ACAs, and then, we compare these results with our archival dataset obtained after injections into the infralimbic/prelimbic region.

## Materials and methods

### Animals

Mice (C57BL/6J) were obtained from JAX and group housed at the California Institute of Technology in a climate-controlled mouse house with a 12-h light–dark cycle. Mice were initially imaged after systemic MnCl_2_ by MEMRI according to our protocol (Uselman et al., 2020). Three weeks after the final scan, projection mapping from ACA was performed Resultant images will ultimately allow comparisons of brain-wide neural activity with anterior cortical projection anatomy. Here, we focus on medial prefrontal forebrain projections. All protocols were approved both at Caltech and UNM IACUC. The numbers of mice were determined by a power analysis based on region of interest measurements before and after MnCl_2_. We used all male mice at an average age of 15.5 weeks (14–19 weeks). We previously compared female and male mice and found no statistically significant differences (Uselman et al., 2020). A second dataset of images from mice injected in the IL/PL regions of the medial prefrontal cortex, which we had previously reported, was retrieved from our archives (Bearer et al., 2009b), aligned with this new dataset, and processed in parallel. These archival images were from 10 female mice, ages 19–23 weeks. We followed the same procedures for the new mice as we had previously reported for the IL/PL injections except for the injection site locations in ACA vs. IL/PL, and the post-injection image collection, where intervals between the first 30 min post-injection image and the final 24 h image differed slightly (Bearer et al., 2009b). To control for any impacts from additional prior experience on the new cohort, we compared behavior at baseline, presumably the status of the non-experienced mice in the archival data, and at 3 weeks after experience, when the intracerebral injections for the new data were performed (Supplementary Figure S1). Exploration was video recorded during the last 10 min of two 30 min periods spent in an arena before (at baseline) and after (23 days, PreFB) experiences just prior to the intracerebral injections. The average time spent not moving at 1-min intervals was tabulated in Ethovision and spreadsheets of values statistically analyzed in R by ANOVA. We found that at the time point of the intracerebral injection (23 days after baseline and 14 days after the last experiences), there was a minor but statistically significant effect on the time spent not moving (*p* < 0.01) of those manipulations as compared to their baseline.

### MnCl_2_ injection

Both datasets were acquired with the same imaging protocol. Mice were anesthetized in 1.5% isoflurane at room temperature. First, after acquiring a pre-injection image (detailed in the following), mice were maintained under anesthesia and mounted into the stereotactic frame. After exposing the skull, a small borehole was drilled, with placement controlled by the stereotactic apparatus to avoid injuring the brain, which in mouse is very close to the skull. The drill was then replaced with a micropipette loaded with 3–5 nL of 0.6 M MnCl_2_ with 0.5 mg/mL of 3 K rhodamine dextran amine (RDA) at 0.5 mg/mL, and 0.15 mg/mL of 10 K fluorescein dextran amine (both from Molecular Probes/Invitrogen/Thermo Fisher) in sterile phosphate-buffered saline. The amount of rhodamine dextran amine molecules was 0.08 pmol or approximately 6.9 × 10^16^ molecules. The solution was injected into the ACA, with the stereotactic device settings of: x, 0.5 mm right of the midline; y, 1.0 mm anterior to bregma; and z, −1.5 deep to the surface of the skull, as described previously (Bearer et al., 2022). Injection was carried out for over 5 min using a hand-pulled quartz micropipette loaded under direct visualization for precise volume control, guided by a computer-assisted stereotactic injector (myNeuroLab.com, IL, United States). After injection, the micropipette was slowly removed, and anesthesia was maintained during the placement of the mouse into the scanner’s mouse holder with its head secured in a Teflon stereotactic unit to reduce movement artifact. Then, the holder was placed into the MR scanner. Within the scanner, anesthesia was maintained (1%–1.5% isoflurane in medical grade air). Inspection of the first image confirmed the injection site placement. Mice were administered 0.3 mL of saline subcutaneously before each imaging session and again upon removal from the scanner. To prevent drying, eyes were treated with ophthalmic ointment before each scan.

### Image acquisition

An 11.7 T 89 mm Bruker BioSpin Avance DRX500 vertical bore scanner (Bruker BioSpin Inc., Billerica, MA equipped with a Micro2.5 gradient system was used to acquire all mouse brain images with a 35-mm linear birdcage radio frequency (RF) coil. Temperature and respiration were continuously monitored during data acquisition with temperature maintained at 37°C and respiration maintained at a rate of 100–120 per minute. A 3D multi-echo RARE imaging sequence combined T_1_ and T_2_ weighting, with a RARE factor of 4, four averages, TR/TE eff = 250 m/12 m; a matrix size of 160 × 128 × 88; and FOV 16 mm × 12.8 mm × 8.8 mm, yielding 100 µm isotropic voxels for a 46-m scan time.

**FIGURE 1:**
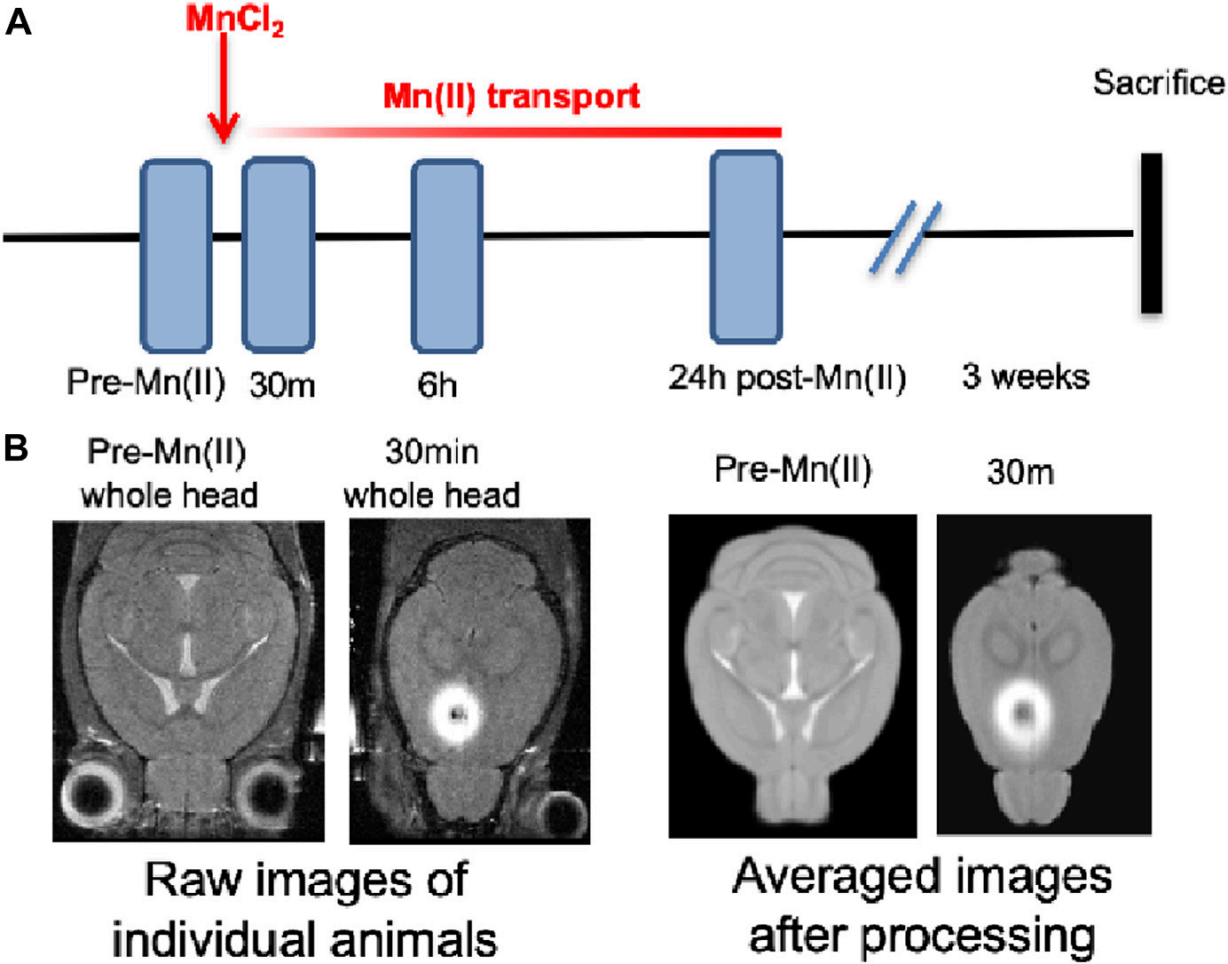
Diagram and examples of experimental procedures. **(A)** Timeline of pre-imaging, MnCl_2_ intracerebral stereotactic injection, time-lapse MR scanning, and euthanasia for histology. **(B)** Slices of MR images at various stages in the processing procedure, from left to right: Raw images of a single animal’s whole head image prior to injection and its 30 min post-injection whole head image shown at a higher axial slice level to pass through the injection site. Both images are taken directly from the scanner and unprocessed. The third and fourth images from the right: Averaged images from the ACA dataset (n = 12) after processing, pre-Mn(II), and 30 min post-injection, shown at analogous slice levels as in the first and second raw images to the left. The averaged pre- Mn(II) image represents the MDT. These second set of images have been skull-stripped, N3 corrected, intensity scaled, and spatially aligned. All images were captured in 3D at 100 µm isotropic resolution.

Each animal underwent four imaging sessions (Figure 1). Images were first captured before MnCl_2_ injection and then at successive time points, beginning immediately after injection (which we will refer to as the 30 min image) and then at 6 h and 24 h, subsequently. Only small inconsequential variations in the exact time after injection were noted. Between sessions, mice were awakened from anesthesia and returned to their home cage to move freely with unlimited access to food and water.

### Histology

After the 24 h scan, mice were returned to the home cage, and at 10–21 days afterward, they were euthanized. This delay was to allow for the slow transport of the dextrans. Each mouse was deeply anaesthetized and perfused with warm heparinized phosphate-buffered saline (PBS; 30 mL) followed by 30 mL of room-temperature 4% paraformaldehyde (PFA) in PBS. The mouse was decapitated, and the head was submerged in 4% PFA in PBS overnight at 4°C. Heads were stripped of skin and hair but maintained in the skull at 4°C in 0.01% sodium azide in PBS for 3 days. After removal from the calvarium, the PBS-azide solution was replenished, and the brains were sent to NeuroScience Associates (NSA, Knoxville, TN) for multi-brain embedding, serial sectioning, and staining. Some sections were mounted directly. for fluorescence imaging of the labeled dextrans. Alternate serial sections of the brains were stained for anatomy by thionine (Nissl).

### Image normalization

All final images used in this study, both new images reported for the first time here and archived dataset, were processed together in a batch. Bruker images were first translated to NIfTI (.nii) files (Neuroimaging Informatics Technology Initiative) and headers matched for all parameters for the uniformity of geometry with FMRIB software library (FSL) (Analysis Group, Oxford, United Kingdom) (Smith et al., 2004; Jenkinson et al., 2012). Voxel sizes, which were originally captured at 100 µm isotropic, were confirmed using the 3drefit program from Analysis of Functional NeuroImages (AFNI) (Cox, 1996). The brain image was extracted from all non-brain areas within the whole head image by masking (a.k.a. “Skull stripping”) according to our standard protocol (Delora et al., 2016). The code for this process is included as Supplementary Data S1 in Delora et al.‘s publication. N3 correction was performed to account for B-field inhomogeneity (Sled et al., 1998; Tustison et al., 2010) on the skull-stripped images in Medical Image Processing, Analysis, and Visualization (MIPAV) (McAuliffe et al., 2001). Images were intensity normalized using our custom script (Medina et al., 2017b). The MATLAB code for this process is included in Supplementary Material S1 with that publication. Our algorithm aligns the modes of each image’s voxel-wise intensity histogram to a single value, calculates, and applies the adjustment needed for all other voxels in the image. Here, we used one of the IL/PL pre-injection stripped images as the reference for scaling. All images in both datasets were then rigid-body aligned to an image randomly selected from the ACA dataset with the “Realign” function in statistical parametric mapping (SPM12) (UCL, London, United Kingdom) (Friston, 1996; Ashburner et al., 2014). Next, a minimum deformation transform (MDT) image was prepared from skull-stripped, modally scaled, pre-injection non-contrast-enhanced images from both datasets by a two step-process. First, images were aligned to an image randomly selected from within the dataset; here, we first selected image from the IL/PL dataset. Resultant images were averaged, creating an averaged image. Then, the same sets of pre-injection images were re-aligned to that averaged image and resultant images averaged again to create the final MDT. For non-linear alignments, we obtained the control point grid (warp field) from the alignment of the pre-injection image to the MDT. The warp field for the alignment of the pre-injection image for each mouse was then applied to that mouse’s post-injection images at all time points after injections using the “Normalize” function in SPM8 (Ashburner et al., 2012). This two-step method avoided potential alignment artifacts that could be introduced during direct warping due to the hyper-intense Mn(II) signal at the injection site (Bearer et al., 2009a). While region of interest (ROI) measurements used the final warped aligned dataset for statistical parametric mapping (SPM) analysis, images were also smoothed with the full-width half-maximum (FWHM) Gaussian kernel set to 0.3 mm using the SPM12 software package (UCL, London, United Kingdom) (Ashburner et al., 2014). Our mouse brain template image (Medina et al., 2017b) was aligned to the MDT using *fsl* fl*irt* (Analysis Group, Oxford, United Kingdom) (Jenkinson et al., 2002; Smith et al., 2004; Jenkinson et al., 2012; Jenkinson et al., 2012). For the ACA injection dataset, we also created an averaged image for each time point from normalized images using FSL.

### Image analysis

While at low concentrations, Mn(II) produces hyper-intense, brighter signal, at high concentrations, Mn(II) signal is hypo-intense, darker, in T_1_-weighted images. Thus, to determine the actual injection site, the hypo-intense region of the high concentration of Mn(II) at the epicenter of the injection site in each animal’s 30 min image was identified, and its 3D slice position was measured in the viewer, FSLeyes, and slice position translated into millimeters relative to the midline (ML), bregma (AP), and brain surface. Our planned injection site for dorsal–ventral (DV, depth) included the skull, since we placed the micropipette on the skull surface to program the stereotactic injector. Hence, our final location, measured from the surface of the brain and not the skull for the DV dimension, is smaller by ∼0.8 mm than the planned injection. The average location of injection sites across the dataset was calculated in Excel. Injection sites were then projected as “landmarks” onto a rendering in 3D of the minimal deformation atlas in Amira (Thermo Fisher).

SPM (SPM12) (UCL, London, United Kingdom) (Ashburner et al., 2014) was used to map Mn(II) enhancements at 6 h and 24 h and to compare maps with archival data from other medial prefrontal cortex injections (Bearer et al., 2009b). Original images from our previous study were retrieved and normalized in our pipeline to align with the new images first reported here. These re-aligned images demonstrated that the archival data had injection sites that were significantly more anterior in the IL/PL areas than the current dataset which was in the ACA. Thus, we first performed within-group comparisons on the new data with ACA-localized injections. Images at 6 h and 24 h were each compared to 30 min and 24 h–6 h using a paired *t*-test design in SPM12. T-values were calculated for *p*-values (0.01–0.0001), uncorrected or corrected for false discovery rate (FDR)` for multiple comparisons. We finally chose a *p*-value threshold based on the most stringent statistic in which some signal was found in all comparisons. We also performed a comparison between 30 min and 24 h of the archival, IL/PL, dataset using a paired *t*-test in SPM 12. An unpaired *t*-test was then performed between 24 h images from each experimental cohort. Maps produced by SPM were visualized in FSLeyes and overlaid on grayscale images, either MDT of the pre-injected images or our standard “muse_template” aligned to these data. As previously described, our *InVivo* Atlas was hand-drawn on this template based on the Allen Institute for Brain Science Mouse Reference Atlas (Uselman et al., 2020). We determined anatomical locations using our most recently updated *InVivo* Atlas (Supplementary Table S1 for atlas segments and abbreviations). The atlas comprised three files, a 16 bit signed gray scale image at 80 µm isotropic resolution, an 8 bit label image at the same resolution, and a lookup table, .lut, which provides the annotations within FSLeyes. The atlas is first aligned to the MDT using fsl flirt, with MDT as the input image and gray scale atlas as the reference image. A 12- parameter affine transformation of the MDT to the gray scale atlas is performed using Search (incorrectly oriented), Cost function (mutual information), Interpolation (nearest neighbor), and with no weighting. In Mac OS14.2 Terminal, we used the fslcommand <convert_xfm> with the inverse. mat file output from flirt to generate an inverse transformation matrix and specify the <inverse affine. mat> function. Then, we first applied the inverse transformation matrix to the gray scale atlas and then to the annotated labeled atlas in *fsl flirt* with <applyxfm>.

### ROI measurements

We used three interdependent custom shell scripts to drive ROI measurements with *fslroi* in FSL (Smith et al., 2004; Jenkinson et al., 2012; Jenkinson et al., 2012). ROI (3 × 3 × 3 voxel cubes) were selected based on the literature as regions receiving input from the mPFC, and coordinates were determined based on evident intensity increases in the average images at 6 h or 24 h and by statistically significant signal in the SPM. Coordinates for each location were entered into the feeder shell script for automated extraction in FSL. Output values were compiled into a single .csv file which was then loaded into R for statistical analysis using the linear mixed model (nlme) and for graphing using ggplot2 (Supplementary Table S2, for ROI coordinates).

### Fractional accumulation volumes and column graphs

To quantify the total number of voxels in each segment, our atlas was applied to the template image and the number of voxels within each segment bilaterally calculated in FSL using the *fslmaths* and *fslstats* functions. To determine the number of enhanced voxels at each time point, individual amoks for each of the 104 sub-regions identified by the *InVivo* Atlas were generated, also through *fslmaths*. Masks were applied to t-maps produced by SPM, using a statistical threshold of *p* < 0.05 FDR (within-group) and *p* < 0.01 FDR (between-group). T-values for this statistic varied: for within group: ACA, 30 min > pre (T = 4.056); 6 h > 30 min (T = 3.912), and 24 h > 30 min (T = 3.573); IL/PL, 30 min > pre (T = 4.786) and 24 h > 30 min (T = 4.809); for between group: ACA > IL/PL (T = 4.05); and IL/PL > ACA (T = 3.60). The ratios of significantly enhanced to total voxels within each segment were calculated in FSL with the *fslstats* function. These ratios were plotted as column graphs with a customized R script.

### Sub-segmentation of the hypothalamus and identification of bregma slice positions

To define sub-segments within the greater hypothalamus not segmented in our current *InVivo* Atlas, we drew segment boundaries identifiable by gray scale contrasts using the Allen Institute for Brain Science Mouse Brain Reference Atlas, histologic anatomy, as the guide. To determine the bregma slice location, we compared anatomy to the Paxinos (Paxinos and Franklin, 2001) using the online interactive site (http://labs.gaidi.ca/mouse-brain-atlas/).

## Results

### Uniform injection locations into the ACA, lack of histologic injury at the injection site, and entry into expected projections

For this study, we aligned newly acquired ACA injections with archival images from our previous publication on MEMRI tract-tracing from mPFC (Bearer et al., 2009b). Then, we measured the position of the injection sites in the 30 min images of living animals from both cohorts as they appeared in this newly aligned matrix. This allowed quantitative comparisons of injection sites between the two cohorts. The average injection site measured across the immediate post-injection images (30 min) from all 12 newly injected animals fell at 0.61 ± 0.11 mm right of the midline, 0.34 ± 0.27 mm anterior to bregma, and 0.82 ± 0.18 mm deep into the brain surface, whereas average locations within the archival dataset were different (Table 1; Supplementary Figure S2). This analysis clearly showed that the two datasets had delivered Mn(II) to slightly different locations in the mPFC. While both sites were close to the right of midline, the new mPFC injections clustered on average 0.84 mm more posterior than those of the archival dataset, which clustered both more anterior and deeper, by 0.49 mm. Thus, the archival dataset fell into IL/PL regions, and the new injections, reported for the first time here, clustered into the ACA. We determined that these differences in injection sites could result in the delivery of Mn(II) to different groups of mPFC neurons which might project to different distal destinations, as has previously been shown by histological tract-tracing (Fillinger et al., 2018). Thus, we segregated our data into two cohorts to pursue this idea. Since we had already published some detail from the archival cohort previously (Bearer et al., 2009b), we first focus on the new data acquired after injections administered into the ACA.

**TABLE 1:** Injection site coordinates.

	Right of center (mm)	Anterior from bregma (mm)	Deep to brain surface (mm)	Largest distance from centroid (mm)
ACA	0.62 ± 0.1	0.34 ± 0.3	−0.81 ± 0.2	1.0
IL/PL	0.56 ± 0.16	1.18 ± 0.2	−1.3 ± 0.3	1.0
PFC (2009)	0.47 ± 0.17	1.1 ± 0.18	−0.88 ± 0.29	ND

PFC, measurements from Bearer et al., 2009 (Bearer et al., 2009b).

**FIGURE 2:**
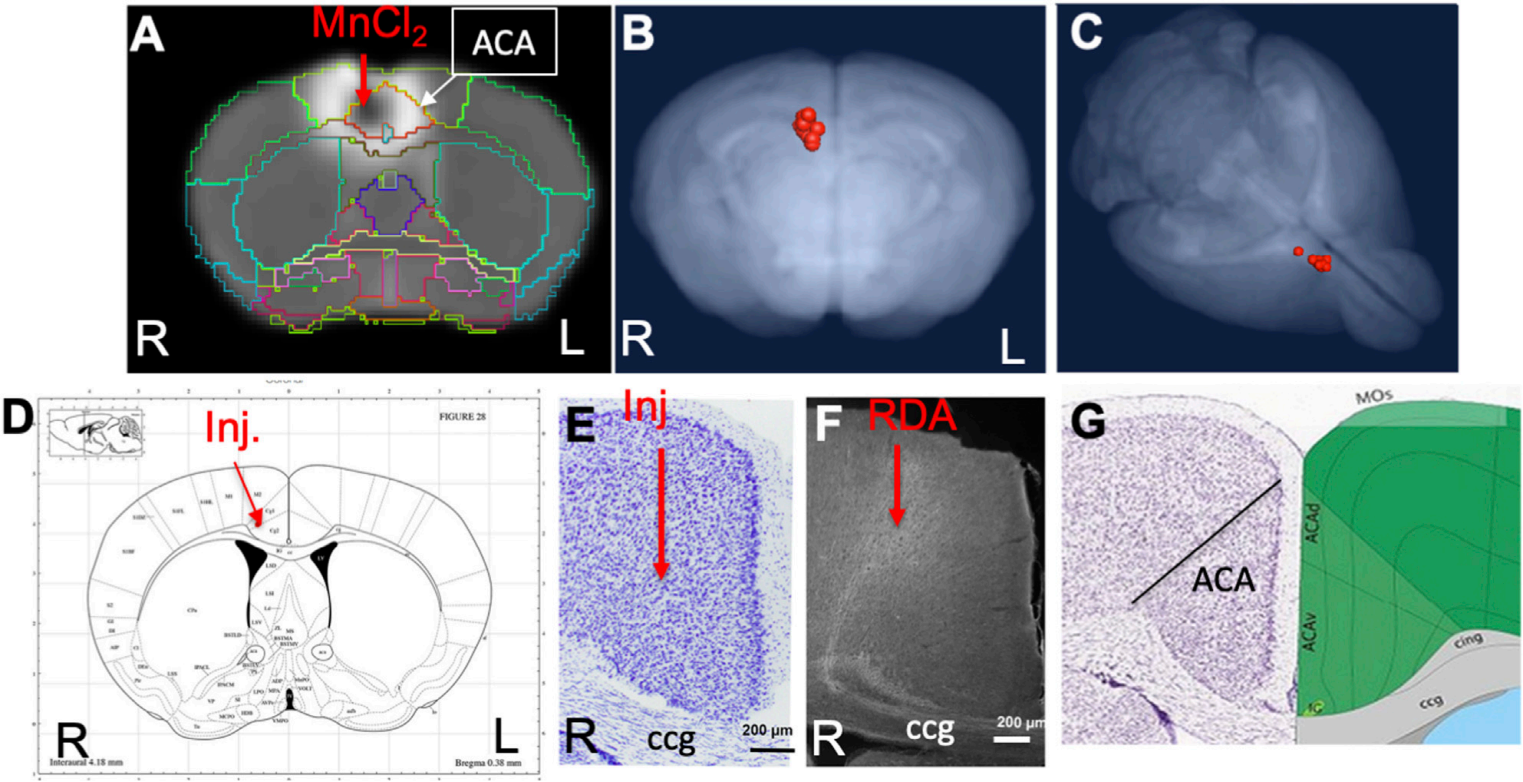
ACA injection site analysis. **(A)** Coronal slice from a single animal with our *InVivo* Atlas segmentation overlaid. Mn(II) high concentration at the center of the injection appears black with a halo of lower Mn(II) concentrations around it. Atlas identifies the injection site in this living mouse as within the ACA. Injection was into the right cortex, with the mouse facing toward the viewer. **(B, C)** Center of the injection sites in all 12 mice projected onto a 3D volumetric image of the mouse brain from our muse-template aligned to this dataset. **(D)** Screenshot from the online Paxinos atlas (http://labs.gaidi.ca/mouse-brain-atlas/) with average coordinates from the MnCl_2_ injection site analysis placed onto the atlas as a red dot. These coordinates appear precisely as in our digital *InVivo* Atlas, within the ACA which in this atlas is labeled Cg1 and Cg2 for cingulate gyrus. **(E)** A section through the ACA from a single mouse in this dataset stained by Nissl. No injection site can be identified. The genu of the corpus callosum is shown (cc). **(F)** Rhodamine fluorescence identifies neuronal processes emanating out of the ACA with no clear injection bolus. Projection goes both into the cortex and down into the fiber tracts of the cingulum bundle and corpus callosum (cc) (Bubb et al., 2018). **(G)** A screenshot of the same region segmented and annotated from the Allen Institute Mouse Brain Reference Atlas. The ACA is sub-segmented, and both cc and cingulum bundle (cig) are labeled (Lein et al., 2007).

The anatomical locations of injections in the ACA dataset were visualized by the overlay of our *InVivo* Atlas on an individual animal’s 30 min post-injection image (Figure 2A). Mn(II) has a biphasic effect on the MR signal, with high concentrations giving a hypo-intense, dark, MR signal appearing in T_1_-weighted images and lower concentrations giving a bright signal appearing as a halo in this 30 min image. The halo extends into adjacent cortical regions, such as the primary motor cortex, but concentrations are unlikely to be adequate to yield statistically significant intensity changes distally from motor neurons. Only the dark region of high concentration hypo-intensity will likely have sufficient Mn(II) uptake for distal mapping. The projection of all 12 injection sites as landmarks onto a 3D image of the MDT further demonstrated consistent placement of these injections (Figures 2B,C). Placement of the coordinates for the average injection location, as determined by our analysis of 30 min post-MnCl_2_ MR images, on the Paxinos online mouse brain atlas (http://labs.gaidi.ca/mouse-brain-atlas/) (Paxinos and Franklin, 2001; Vogt and Paxinos, 2014) showed the injection site fell within the ACA, labeled as CG1 and CG2 according to Paxinos annotations in this online interactive atlas (Figure 2D).

Injection sites were not detectable histologically, as no evidence of long-term injury could be found in Nissl-stained serial sections from animals fixed at 2–3 weeks after injection, as previously reported (Figure 2E) (Bearer et al., 2009b). Only by searching for co-injected dextran fluorescence across serial sections were injection sites identified histologically (Figure 2F). RDA was identified in a region consistent with the ACA, with the fluorescent tracer transported along apparent axons into the corpus callosum (cc) and cingulum bundle (cig), regions identified according to visual comparisons with the Allen Institute Mouse Brain Reference Atlas (Figure 2G) (Lein et al., 2007). These local processes resembled those reported in individual mice traced by fluorescent tracers in the connectome project by the Allen Institute for Brain Science. The microscopic evaluations of the ACA-injected brains also attested to minimal injury and accurate placement into the ACA.

### Mn(II) enhancements visualized in averaged 3D images after injection into the ACA

**FIGURE 3:**
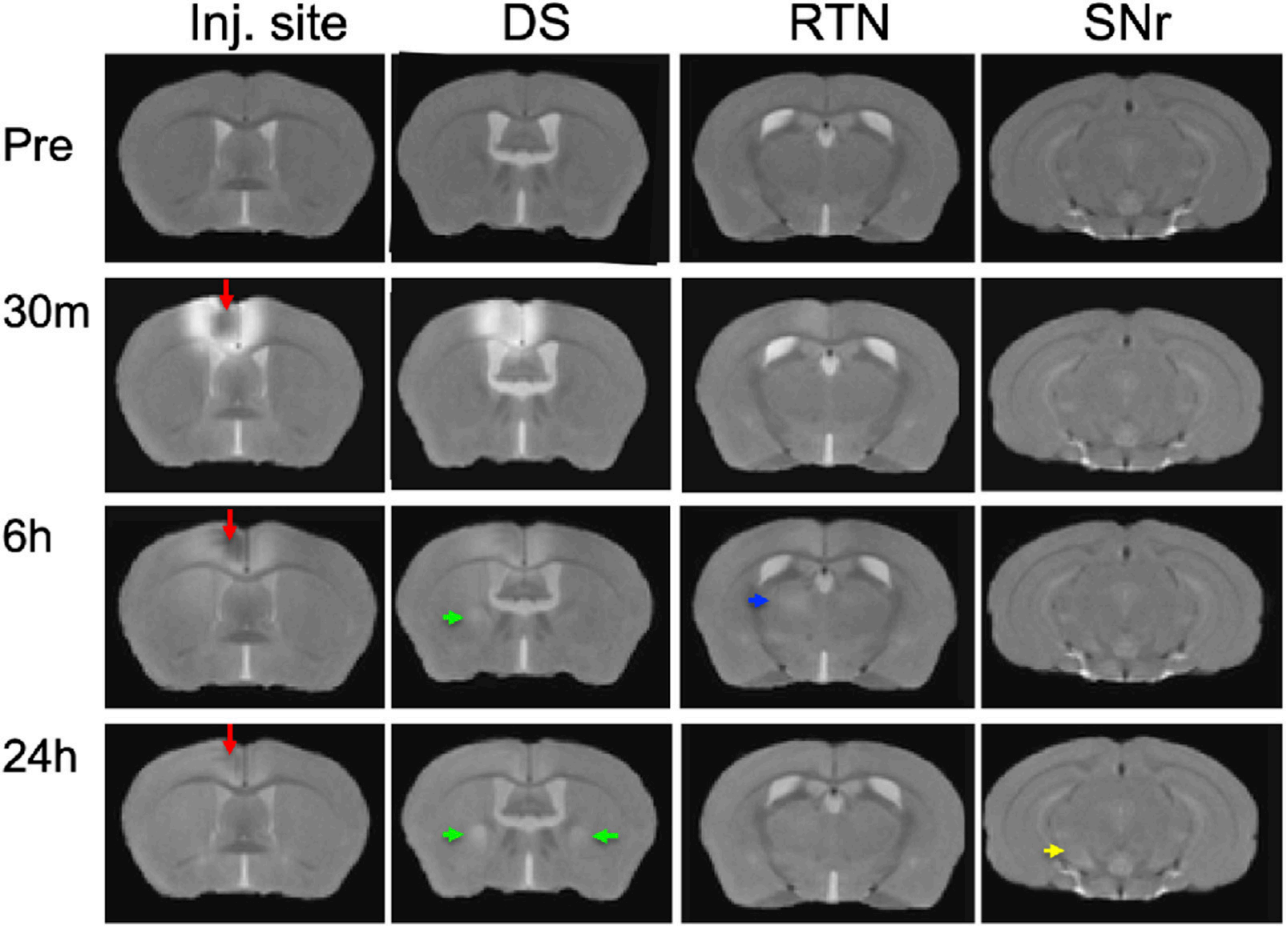
Mn(II) enhancements appear in multiple regions across slices at each time point. Coronal slices were selected at four anterior–posterior locations from the averaged aligned 3D images of the twelve animals at each time point. Arrows indicate hyper-intense signals: *red*, injection site; *green*, dorsal striatum; *blue*, reticular nucleus of the thalamus; and *yellow*, substantia nigra reticulata. Anatomy was confirmed by reference to our digital *InVivo* Atlas.

To ascertain that Mn(II) had successfully progressed into the brain, we averaged the aligned images from each time point and inspected coronal slices for intensity increases in expected locations. Whole brain images captured in living animals by time-lapse MRI were aligned to the pre-injection MDT, and then, the subset of images for each time point (pre-injection and 30 min, 6 h, and 24 h post-injection) across the 12 animals were averaged (Figure 3). Here, we show coronal slices from these averaged 3D images at each of the four time points, with slice position selected to pass through the expected target locations of ACA projections. Mn(II) enhancements were readily apparent in these slices and followed expected ACA projections: dorsal striatum (DS), reticular nucleus of the thalamus (RTN), and substantia nigra reticularis (SNr). In this inspection, we also see the differences in time at which enhancement appears in each of these distal targets, in ipsilateral DS at 6 h and in both ipsilateral and contralateral DS at 24h, in the contralateral RTN at 6 h where it then fades at 24 h, and finally reaching SNr by 24 h, possibly bilaterally. Also apparent is the decrease of Mn(II) intensity over time in the injection site, where it virtually disappears by 24 h. These visual inspections are not sensitive enough to detect all the projections of the ACA, the dynamics of accumulations between time points, or the degree or statistical significance of signal enhancements.

### Statistical parametric mapping of Mn(II) intensity enhancements after ACA injection

**FIGURE 4:**
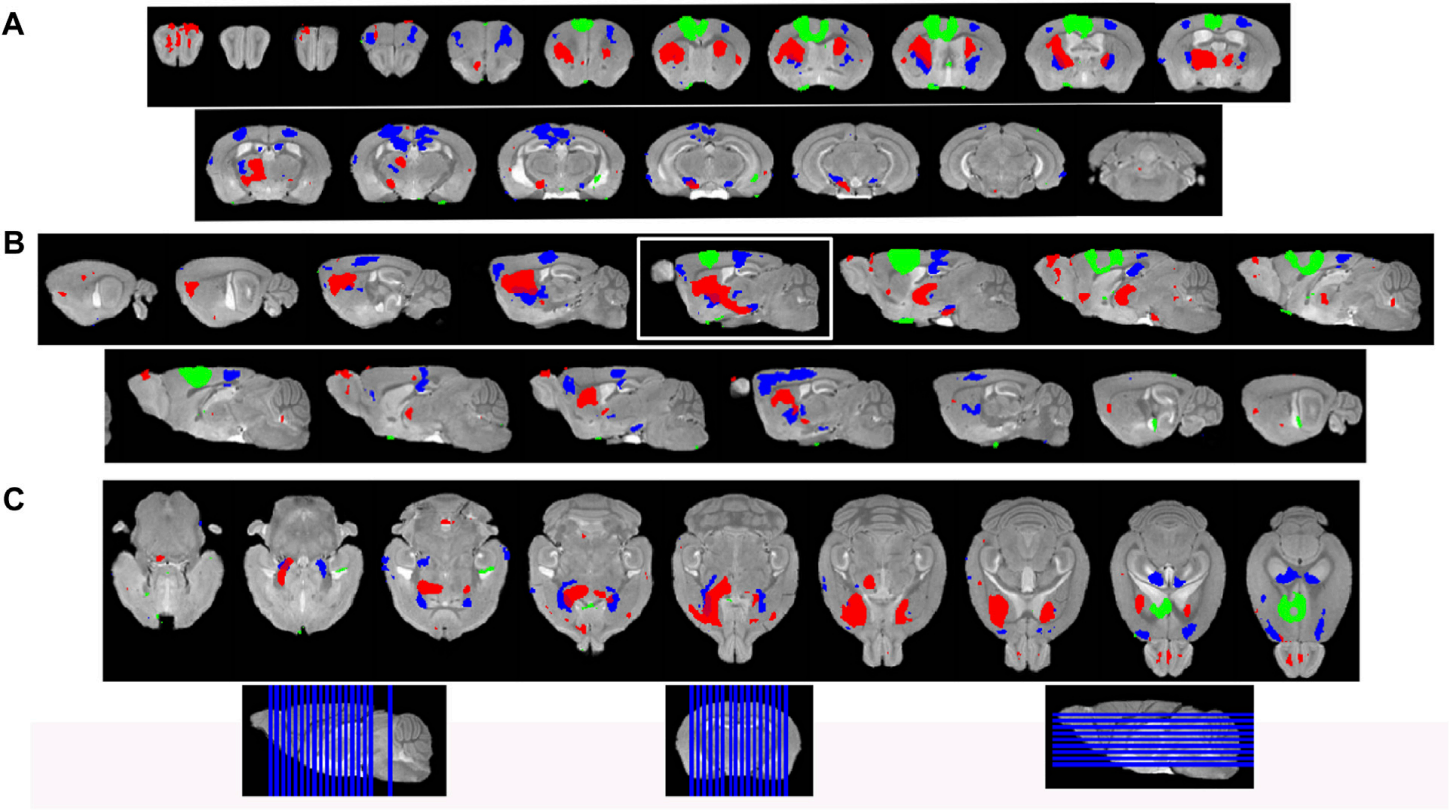
Statistical parametric mapping shows progress of Mn(II)-dependent intensities across time points. **(A)** Coronal slices of SPM maps from each time point overlaid on the high-definition template shown from left to right in the figure from anterior to posterior at 0.5 mmm intervals. **(B)** Sagittal slices at 0.5 mm intervals taken from slices right to left of the brain shown going left to right in figure. **(C)** Axial slices from ventral to dorsal at 0.5 mm intervals, shown from left to right in the figure. Green, 30 min > pre-Mn(II); red, 6 h > 30 min; and blue, 24 h > 30 min. Slice positions are diagrammed at the bottom of the figure on each orientation of the whole brain 3D image.

To increase sensitivity and identify regions brain-wide that received significant Mn(II) after localized injections in the ACA at each progressive time point, we adopted statistical parametric mapping (SPM) (Bearer et al., 2007b). Such computational approaches detect statistically significant voxel-wise intensity changes in an unbiased and brain-wide comprehensive manner which has broken open our ability to understand human brain dynamics from BOLD MR imaging. When applied to MEMRI, SPM reveals where Mn(II) has had a significant impact on the MR signal (Figure 4). SPM shows that Mn(II) enhancements progressed from the injection site into the contralateral cortex, as could be seen in MR images, and traveled deeper into subcortical regions. At 6 h, shown in red, a statistically significant Mn(II) signal occurred in the DS and tracked down, as seen best in the sagittal slice outlined in white, trafficking all the way to the SNr in the brainstem. From 6 h to 24 h, the Mn(II) signal transported even deeper into these structures and also appeared in the retrosplenial cortex and dorsal hippocampus. While this cortical signal could be dismissed as diffusion from the injection site, the lack of signal at 6 h (red) between the 30 min signal (green) at the injection site and the 24 h (blue) suggests that the 24 h signal in the retrosplenial cortex is at least partly produced, not by diffusion from the injection site, but by transport either along the corpus callosum from one cortical region to another or indirectly from a polysynaptic circuit first to the DS or RNT and then back up to the cortex progressing toward the hippocampus.

We then took two additional steps to parse the segmental distribution of transported Mn(II), thereby obtaining clues about the ACA projection anatomy: 1) measuring intensity increases with region of interest analyses; 2) quantifying the volume of enhanced voxels, the fractional accumulation volume, in every segment across the brain by applying our *InVivo* Atlas to enumerate statistically enhanced voxels and obtain the ratio of enhanced to total voxels. Finally, we moved on to compare fractional accumulation volumes, the volume of Mn(II) enhancement within each segment, between ACA and the IL/PL injection sites with two-tailed unpaired statistical models.

### Mn(II) transports along predicted pathways after ACA injection by region of interest analysis

**FIGURE 5:**
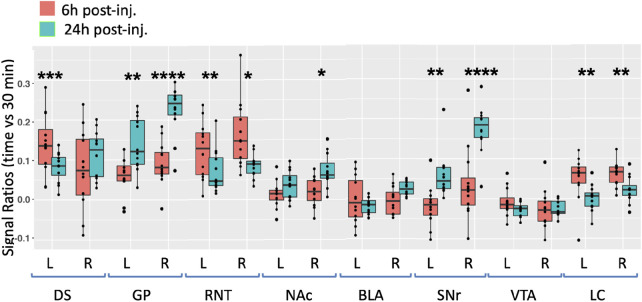
Degrees of intensity changes in regions of interest over time after ACA forebrain injection. Measurements of intensity values in 3 × 3 × 3 voxels selected in the dorsal striatum (DS), globus pallidus (GP), reticular nucleus of the thalamus (RNT), nucleus accumbens (NAc), basolateral amygdala (BLA), substantia nigra reticulata (SNr), ventral tegmental area (VTA), and locus coeruleus (LC). Both contralateral (left, L) and ipsilateral (right, R) were measured in the 30 min, 6 h, and 24 h images. Intensity values were calculated as a ratio at 6 h (orange) or 24 h (green) over that of the 30 min images. Box and whisker quartile plots are shown with individual values projected as scatter plots. Some median values increased between 6 h and 24 h and others decreased. Outliers greater than 2 standard deviations are shown in the scatter plot and included in the statistics. Statistical significance of comparisons using the linear mixed model between 6 h and 24 h intensities regardless of directionality as indicated: *p < 0.01; **p < 0.01; ***p < 0.0001, and ****p < 0.00001.

To quantify Mn(II) distal accumulations over time in the ACA injection group, we selected eight cubic regions of 3 × 3 × 3 voxels bilaterally which were predicted by previous reports to receive input from the ACA, and measured average intensity values (Figure 5). Measurements were made in 30 min, 6 h, and 24 h images across the ACA dataset. Segments were identified with our *InVivo* Atlas. Signal increases at subsequent time points (6 h and 24 h) were determined as the ratio of signal intensity in the same ROI divided by intensity in the 30 min image. We reasoned that any signal distal to the injection site at 30 min after injection was more likely due to rapid interstitial diffusion of Mn(II) than to slower membrane-bound vesicular transport (see Supplementary Table S3 for ROI coordinates, Supplementary Table S3 for measurements, and Supplementary Table S4 for statistics).

At 6 h after ACA injections, the signal increased more than 10% in both the left and right DS and RNT and on the right GP, while the locus coeruleus (LC) bilaterally had >5% intensity increases. We compared increases from 6 h to 24 h statistically and found significant increases in both sides of the GP and SNr and in the right NAc ipsilateral to the injection site. A small increase of a few percent was also detected in the right BLA at 24 h, which was not statistically significance. These increases likely represented continued transport to these regions, possibly secondarily from accumulations at other targets within a polysynaptic projection anatomy. In contrast to the increasing signal at 24 h in these ROIs, the signal decreased significantly in a few others compared to the 6 h time point, with decrease on both sides of the RNT and LC. These decreases together with the loss of the signal in the injection site over time, as shown in Figure 3, may represent less Mn(II) entering the transport system from projections that directly synapse on these segments at later time points.

### Projection fields differ in ACA injections compared to IL/PL

**FIGURE 6:**
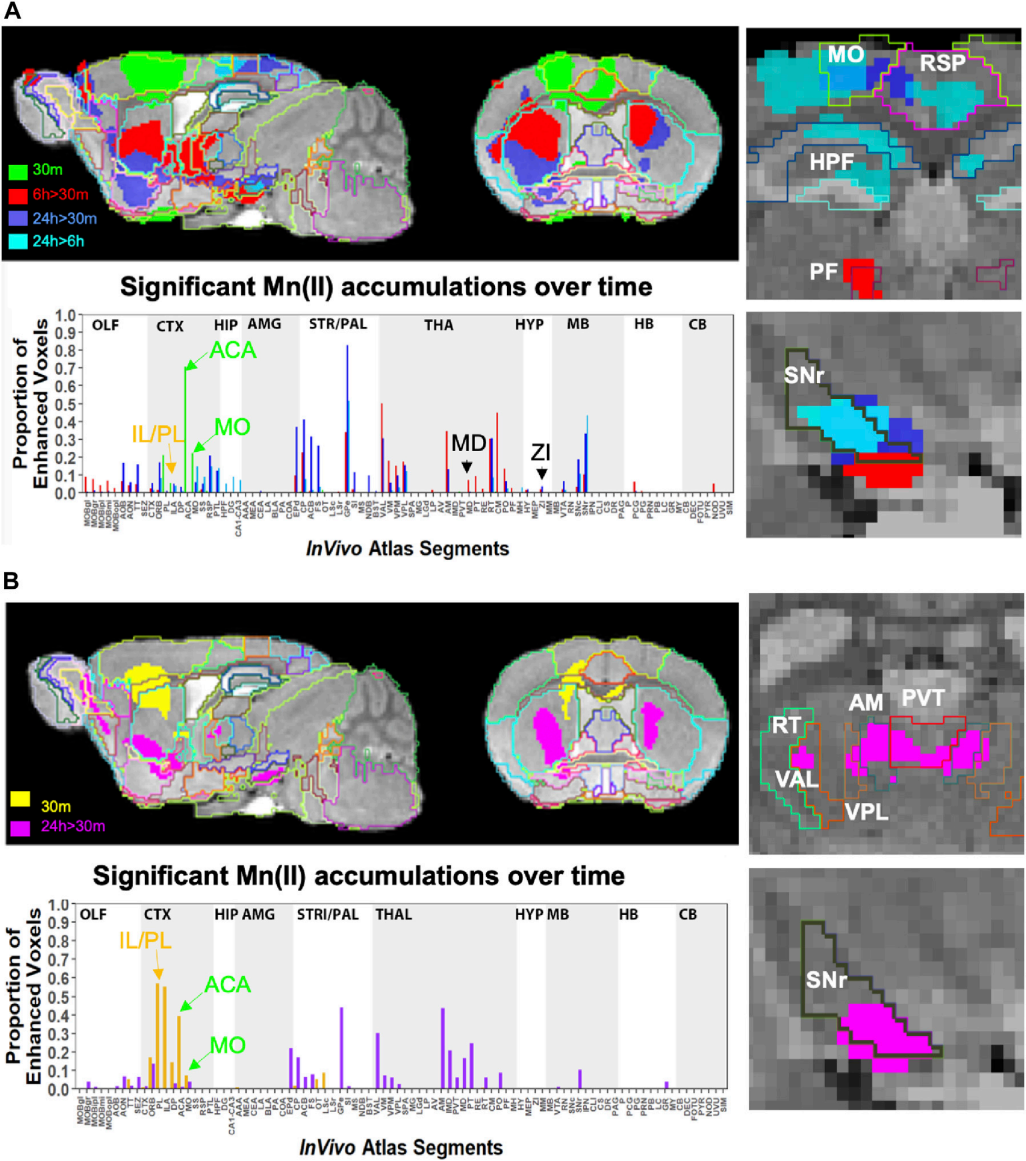
Comparison of projection maps from ACA with IL/PL by visual inspection of within-group statistical maps and segment-wise fractional accumulation volumes. **(A)** Slices from voxel-wise brain-wide maps of the anatomy of statistically enhanced voxels after ACA injection projected onto a gray scale image of our high-definition template are shown: Green, 30 min > pre-Mn(II) (T = 5.42); Red, 6 h > 30 min (T = 5.26); Blue, 24 h > 30 min (T = 6.01); and Turquoise, 24 h > 6 h (T = 4.88) (*p* < 0.05 FDR for all comparisons). In this overlay, images are layered on the template from bottom to top in the following order: 24 h > 30 min, 6 h > 30 min; 30 min > pre-Mn(II); and 24 h > 6 h with the *InVivo* Atlas v10.2 segmentation delineated on the top layer. Right two panels show enlargements of coronal slices: Time of the statistical map color-coded, with red, 6 h > 30 min, dark blue, 24 h > 30 min, and turquoise, 24 h > 6 h. Segments outlined in color in the top right panel: Retrosplenial area, RSP, hot pink; motor cortex, MO, chartreuse; hippocampal formation, HPF, dark green; parafascicular nucleus of the thalamus, PF, red; and coronal slice, upper enlargement, coronal slice 83. Right panels, second from the top: substantia nigra reticulata, SNr, black; coronal slice 94. *Lower left panel*, column graphs showing fractional accumulation of Mn(II) signal segment-wise calculated as the fraction of enhanced to total voxels within each of 110 segments at this T value. **(B)** Similar overlays as **(A)** but after IL/PL injections: Yellow, 30 min > pre-Mn(II) (T = 4.78); Magenta, 24 h > 30 min (T = 4.8) (*p* < 0.05 FDR for both comparisons). Lower right two panels show enlargements of coronal slices with SPM of 24 h > 30 min in magenta. Bilateral segments are outlined in color: Anteromedial nucleus of the thalamus, AM, olive; ventral anterior lateral thalamic complex, VAL, red-brown; intermediodorsal nucleus of the thalamus, IMD, brown); ventral posteromedial nucleus of the thalamus, VPL, dark blue; paraventricular nucleus of the thalamus, PVT, red; and coronal slice, 74. *Lowest right panel*: Substantia nigra reticulata, SNr, black; coronal slice, 94. *Lower left panel*, column graphs showing fractional accumulation of Mn(II) signal segment-wise calculated as the fraction of enhanced to total voxels within each of 110 segments at this T value.

To pursue the possibility that these minor differences in forebrain injection sites might trace different anatomical projections, we first performed voxel-wise paired t-tests by SPM on each aligned dataset separately and compared results between groups by visual inspection (Figures 6A, B, upper panels). Differences in the placement of injection sites were evident in sagittal slices when 30 min time point images are overlaid: the ACA, shown in 6 A in green; IL/PL in 6 B in yellow (Supplementary Figure S2). Next, we calculated the fraction of each segments’ voxels that was statistically enhanced and visualized results in column graphs (Figures 6A, B, lower panels). In segments within the injection site in the ACA cohort, there was a large ratio (0.7) of enhanced to total voxels in the ACA segment and only a low ratio of enhanced to total voxels within IL/PL segments (Figure 6, lower panels, arrows). In contrast, in the IL/PL cohort, the ACA segment has less volume occupied by statistically enhanced voxels (0.4), and both IL/PL segments had a 0.6 ratio of enhanced voxels to total. In the ACA cohort, some spill over into the motor cortex (MO) (0.2 fraction of total voxels) was present, although little to no signal appeared in the mediodorsal nucleus of the thalamus (MD) or the zona incerta (ZI), direct projections from the primary motor cortex (Gan et al., 2022) (Supplementary Figure S3). At 6 h in the ACA cohort (Figure 6A Top panel, red), SPM maps showed the progression of significantly enhanced voxels down through the brainstem as far as the SNr with a track that appears distinct, although somewhat also diffuse in both groups. A similar track has been detected in reassembled whole brain sections after viral vector tracing from the ACA (Shi et al., 2022) and may represent the cerebral peduncle, which was omitted from our *InVivo* Atlas along with other fiber tracks when quantifying fractional accumulation volumes (SPM maps in Supplementary Figure S3).

Column graphs at 6 h and 24 h time points in the ACA group show fractional accumulation volumes increased in some but decreased in other segments between 6 h and 24 h (Table 2). Since for the IL/PL group, we only have 30 min, 1 h 40 min through 4 h 20 min, and a final 24 h post-injection images, we could only compare the 30 min and 24 h images between the two injection groups (Bearer et al., 2009b). It is tempting to speculate that differences in accumulation rates reflect direct and indirect trans-synaptic projections, with the striatum and thalamus receiving the earliest arrivals and other regions more delayed due to being secondary connections. This interpretation may be most reliable for the accumulations from 6 h to 24 h, turquoise color coded in Figure 6A, particularly those in the motor cortex and hippocampus. However, since we do not have enough information about the rate of Mn(II) transport, time to cross a synapse, nor the number of synapses in these projections, this speculation will require further study.

**TABLE 2:** Timing of accumulation volumes after ACA injection.

Segments with greater volume of accumulation at 6 h *versus* 24 h	Segments with greater volume of accumulation at 24 h *versus* 6 h	Segments with similar volumes at both times	Segments with accumulation 6h–24 h
VAL	APN	VPL	ORB
VM	TT	RT	MO
VPM	RSP		SS
AM	PTL		RSP
PF	EPd		HPF
PCG	CP		CA1-3
NOD	ACB		DG
	FS		SNr
	GPe		
	VTA		
	SNc		
	SNr		

Comparisons of statistical maps and fractional accumulation volumes between the two injection groups at 24 h showed Mn(II) enhancements had progressed further in the ACA compared to the IL/PL group (Figure 6A, blue compared to 6B, magenta, in both maps and columns), with greater accumulation and more posterior progression in the ACA cohort. In contrast, in the IL/PL cohort, there was less progress into segments within the midbrain (MB) or hindbrain (HB) at 24 h. These SPM and column graphs of fractional accumulation volumes extend the information gleaned from ROI analysis by detecting sub-segmental dynamics and identifying additional regions not previously recognized and, thus, not selected *a priori* as candidate regions for ROI analysis. Such additional segments in the ACA cohort include AOB in the olfactory system; EPd in the amygdala; CP and FS in the striatum/palladium; RSP in the cortex; VAL, AM, and CM in the thalamus; and small volumes of statistically enhanced voxels in the hindbrain (PCG) and cerebellum (NOD). A subset of these also appear in the IL/PL analysis In comparison, the IL/PL injected cohort seemed to have overall less distal accumulation, suggesting lower transport, although the total number of enhanced voxels in the forebrain was similar in both cohorts testifying that the amount of Mn(II) injected was equivalent. Differing distal destinations could obscure some greater accumulations in IL/PL as segment volumes are not uniform. Although small alterations in experimental procedures between individuals within the cohorts could have inadvertently occurred, any effect from this possibility would be minimized by our sample size, 10–12 animals in each cohort, and statistical analyses. While mice in IL/PL were all female, those in the ACA were all male, partly due to changes in IACUC regulations, although both groups were of the same strain and similar ages. The ACA mice had also undergone additional manipulations while at Caltech not experienced by the IL/PL injected cohort.

Many similarities and a few differences in segmental accumulations appeared in visual comparisons between segment-wise column graphs. The most evident difference in addition to the injection site was more transport into the midbrain after ACA injection and possibly more to the thalamus after IL/PL injection. These visual inspections were not as satisfying as a direct statistical comparison by unpaired t-tests between the two groups.

### Between group analyses reveal dramatic, statistically significant differences in distal accumulations from different mPFC injection locations

**FIGURE 7:**
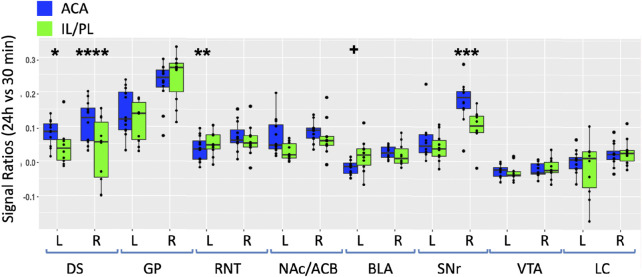
Between-group comparisons of the region of interest measurements of distal accumulations from either ACA or IL/PL injections. Box and whisker quartile plots are shown with individual values as scatter plots. Intensities were measured at 30 min and 24 h time points between the two groups in the same ROIs shown in Figure 5, as indicated (blue, ACA data; green, IL/PL data). Ratios of 24 h *versus* 30 min were calculated and compared using a linear mixed model.+*p* < 0.11; *p < 0.01; **p < 0.001; ***p < 0.0001; and ****p < 0.00001.

**FIGURE 8:**
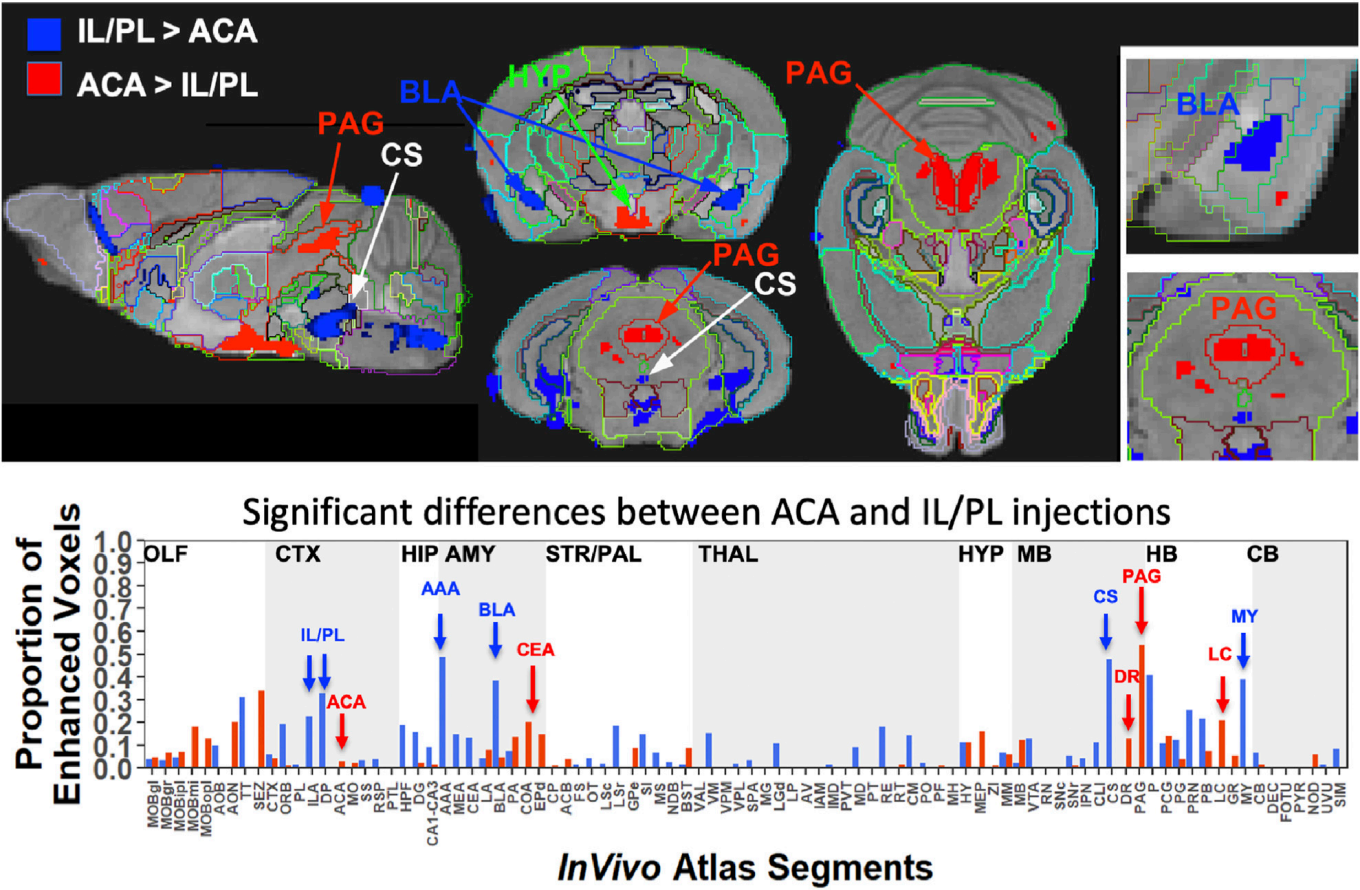
Between-group statistical parametric mapping and segment-wise difference in volumes of Mn(II) accumulations. Two sample t-tests between the two groups at 24 h after injection in SPM produced two different maps (top panels). T-maps are overlaid on the gray scale MDT with *InVivo* Atlas superimposed as outlines. Red, ACA greater than IL/PL, *p* < 0.001 FDR, T = 5.6, and blue, IL/PL greater than ACA, *p* < 0.001 FDR, T = 5.1. Column graphs of the fractional difference volumes (lower panel) identify major increases in the volumes of statistically significant voxels after ACA injections (red) in the PAG, dorsal raphe (DR), and locus coeruleus (LC) and increases after IL/PL injections (blue) in the amygdala, particularly the anterior amygdala area (AAA) and basolateral amygdala (BLA), as well as the central superior raphe nucleus (CS) and medulla (MY), which is not extensively subdivided in this atlas. While statistically significant voxels increase in IL/PL segments in that group compared to the ACA group, only a minor increase in the fraction of enhanced voxels in the ACA segment is found in ACA group over IL/PL group. Because the high-concentration centroid of the injection site is hypo-intense, this could decrease the number of enhanced voxels found in those segments receiving the injection.

For a direct comparison between groups, we first measured the same ROIs, as shown in Figure 5 in both ACA and IL/PL groups (Figure 7). We chose the 24 h time point to compare, as that was captured at nearly identical times after injection in both groups. We calculated the ratio of an average MR intensity signal at 24 h *versus* 30 min in each group and performed statistical comparisons between groups. In four of the eight segments, measured bilaterally, the differences between groups were significant, with the ACA greater than IL/PL in the DS bilaterally and in the ipsilateral (right) SNr and with IL/PL significantly greater than the ACA in contralateral (left) RNT and marginally significantly greater in left BLA as compared to left BLA in the ACA. These results led us to perform a voxel-wise brain-wide unpaired *t*-test between the two groups with SPM and then calculate fractional accumulation volumetric differences (Figure 8).

This SPM analysis was performed in two directions, with IL/PL greater than ACA injections and *vice versa*. Resultant SPMs detected voxels with significant intensity differences between the two cohorts. Even at a fairly high stringency threshold of *p* < 0.001 FDR corrected (T = 5.6 for ACA and 5.1 for IL/PL), significantly different voxels within specific segments were highlighted. Segments with different volumes of significantly different intensity values were found within specific brain regions previously implicated in different mPFC functions, such as amygdala, hypothalamus, and the monoaminergic systems. Increased volumes of Mn(II) accumulations occurred in different segments depending on the injection site (Figure 8, top panel). By projecting both of these between-group SPM maps at the same *p*-value onto our *InVivo* Atlas we found that segments with the largest volumes of significantly enhanced voxels that were also significantly different after IL/PL injections compared to those from the ACA were basolateral amygdala (BLA) and central superior raphe (CS), whereas those less enhanced after IL/PL injection and more so after ACA injection were in the posterior hypothalamus (HYP) and periaqueductal gray (PAG) (Figure 8, lower panel; Table 3). These images only represent significant differences between these two mPFC projection fields; they do not represent the full trajectory of projections from those two injection sites, as segments that are similarly enhanced between them will not be detected by this type of comparison. Analysis of the volumes of the significant differences of statistically enhanced voxels per segment was performed, and the results were visualized as column graphs (Figure 8, lower panel). In this case, volumes of enhanced voxels were extracted from the between group unpaired two-tailed SPM t-maps, with the threshold set at *p* < 0.01 FDR (ACA, T = 4.05; IL/PL, T = 3.6), a lower stringency compared to the SPM maps shown in the top panel. Many additional locations with 10%–15% differences in active volumes were found.

**TABLE 3:** Differences in Projections from ACA and IL/PL Anatomical differences are based on between-group two-tailed SPM *t*-test and correlations to our *InVivo* Atlas.

	ACA	IL/PL
Domains	Segments	Segments
Amygdala	CEA	AAA
	EPd	BLA
Midbrain	PAG	CS
	DR	
Hindbrain	LC	*p* (Pons NOS)
		MY (Medulla NOS)
Hypothalamus	DVH	AHN
	PVp	LHA
	MOB	

In small segments where even a small amount of signal would occupy a large ratio of voxels, this analysis matched visual inspection of the maps. However, in some other segments with a larger number of total voxels, these calculations did not seem to reflect what was visible in the SPM maps. This issue was particularly troublesome in the hypothalamus, where localized differences between the two groups could be seen when scrolling through the 3D dataset but little difference appeared in the column graphs, and could affect other segments as well. Hence, we assumed that the large number of total voxels in the hypothalamic segment of our *InVivo* Atlas overwhelmed the localized signal from differing sub-segments within the greater hypothalamus during ratiometric calculations.

**FIGURE 9:**
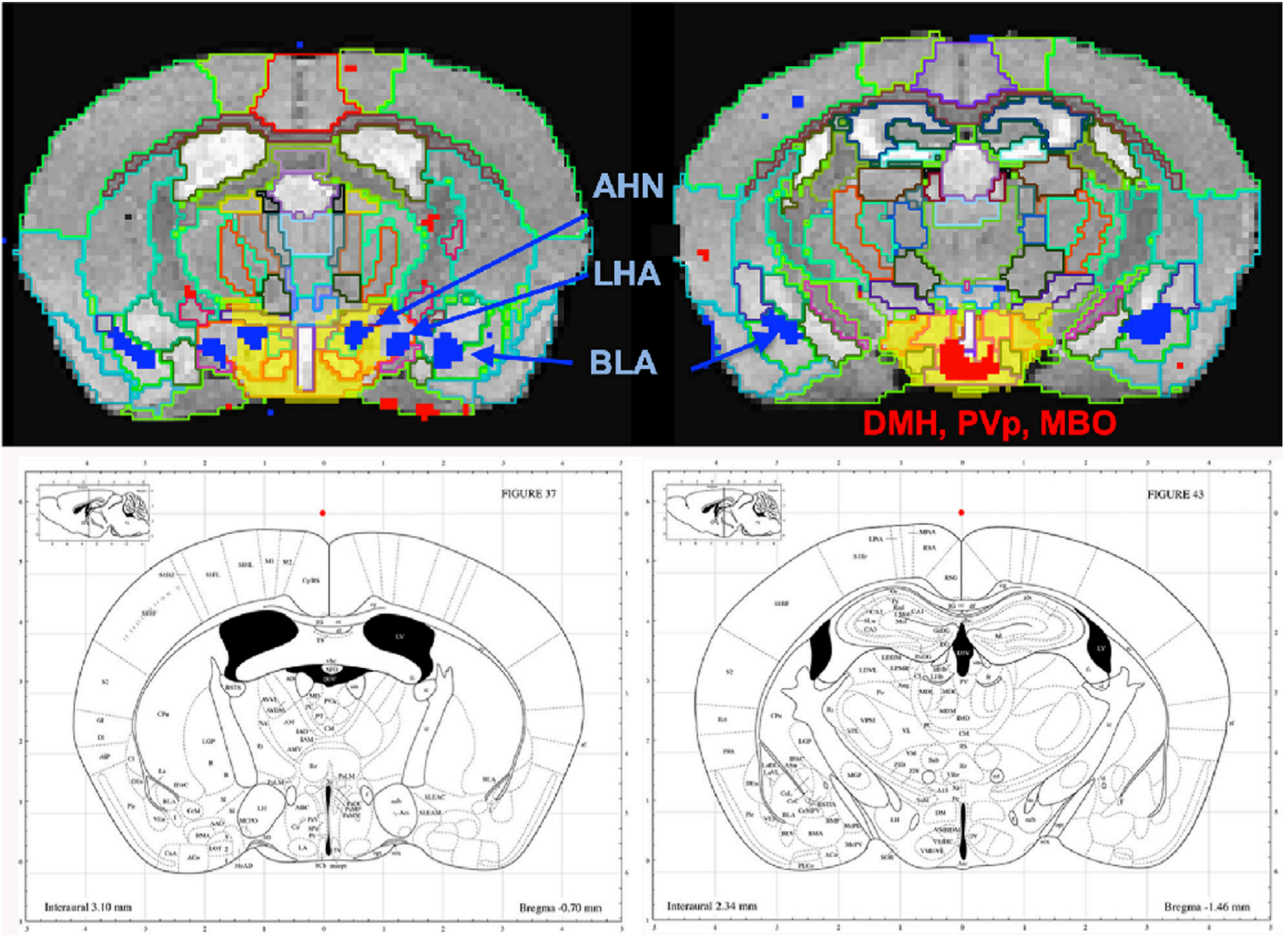
Differential accumulations of Mn(II) transported to the hypothalamus after IL/PL or ACA injections. Coronal slices at higher magnification of overlays of the two-sample t-tests shown in Figure 8 onto a gray scale high-contrast muse-template image with sub-segmentation of the hypothalamus at coronal slices: A-P, 72 (left) and A-P, 81 (right), 900 µm more posterior, with IL/PL > ACA (blue), and ACA > IL/PL (red). The full hypothalamic segment is colored yellow (top two panels). This segmentation was used to generate the segment-wise fractional difference volumes for the column graphs shown in Figure 8. Here, the hypothalamus is subdivided into the anterior hypothalamic nucleus (AHN), lateral hypothalamic area (LHA), dorsomedial nucleus of the hypothalamus (DMH), periventricular hypothalamic nucleus, posterior part (PVp), and mammillary body (MBO), as indicated. Screenshots of the online interactive Paxinos atlas at these slice positions (lower panels) (http://labs.gaidi.ca/mouse-brain-atlas/) at bregma - 0.70 (left lower panel) and - 1.49 (right lower panel) are shown (Paxinos and Franklin, 2001, Access online via Elsevier, 2001. A tool by Matt Gaidica).

To dive more deeply into possible differences between projections within the hypothalamus from these two sites, we sub-segmented this region on a high-resolution, high-contrast MEMRI image with anatomy based on the gray scale high-contrast image with reference to both the Allen Institute Mouse Brain Reference Atlas (Lein et al., 2007) and Paxinos (Paxinos and Franklin, 2001; Vogt and Paxinos, 2014) (Figure 9), which are histologic atlases. Boundaries between smaller subdomains within the hypothalamus can be difficult to define in the gray scale MR image even at high contrast and resolution. However, having an MR-based digital atlas aligned to our dataset greatly facilitated segment-wise analysis and was a major improvement over visual comparisons between MR images and histologic atlases. After computational inverse alignments of this sub-segmented digital atlas to our SPM maps, we could easily observe dramatic differences in accumulations within specific identifiable sub-regions of the hypothalamus, any one of which could have major functional implications. The IL/PL injected cohort showed statistically significant bilateral accumulations in the anterior hypothalamic nucleus (AHN) and lateral hypothalamic area (LHA) (Figure 9, blue), as well as BLA, previously shown in Figure 8, whereas the ACA (Figure 9, red) had more accumulation compared to IL/PL in other regions, with significantly different voxels clustering in the dorsomedial nucleus of the hypothalamus (DMH), periventricular hypothalamus posterior part (PVp), and mammillary body nuclei (MBO). While IL/PL projections accumulated Mn(II) signal more in anterior regions, approximately to bregma - 0.7, ACA projections reached more caudally, approximately to bregma - 1.48. Slice locations relative to bregma were determined by matching MR morphology with the online Paxinos atlas (http://labs.gaidi.ca/mouse-brain-atlas/) (Figure 9, lower panels). This represents a dramatic difference in average projection fields from these two closely placed forebrain regions, less than a millimeter apart anatomically, both in their targeting to PAG, a site of arousal, or to the BLA, a site for fear processing, and to different sub-regions within the greater hypothalamus where brain activity is translated to hormonal release.

## Discussion

By employing MEMRI to trace projections from mPFC in living animals after injection of tracer into two adjacent but distinct locations, we add to the growing literature about functional sub-domains within this region. Here we report that tracer injected into the ACA preferentially transports to posterior hypothalamic nuclei, mammillary nuclei, periaqueductal grey, central nucleus of the amygdala, endopiriform nucleus, dorsal raphe and locus coeruleus as compared to the same tracer injected into the IL/PL, less than a millimeter away, which transports to more anterior segments in the hypothalamus, both anterior and basolateral amygdala, central raphe and various non-annotated discrete regions in pons and medulla. Thus, in mouse the two regions have distinct distal connections and may thus be capable of regulating varying limbic system responses.

This work is unique in a number of ways. By standardizing the injection sites, we traced projections from cohorts of 10–12 mice and report here average results across all animals in each cohort. No sacrifice was necessary, as transport was imaged in the living animal over a short, 24 h, time period. By capturing images during transport and following progress in the living brain over time, we found that transport from a bolus of tracer could arrive at distal destinations within 6 h, and may either increase into that target or dissipate. Thus, these data reveal that accurate distal projection mapping depends on precise injection site, and both on transport and clearance. Timing between tracer injection and imaging may be critical. Because Mn(II) even at this low dose gives a robust signal and we have an annotated digital atlas with a computational processing pipeline that allows us to average across all images in both cohorts with 22 mice and three to four images for each, our data is sufficient to gather statistically significant results. By measuring intensity values across all animals in candidate regions-of-interest, we obtain values for variance, average intensity changes, and statistically significance differences between time points both within the ACA group and between the two groups. This analysis is in part made possible by the anatomical uniformity of the C57BL/6 mouse and by our computational intensity normalization and alignment procedures. The type of information derived from this study has never been available until now.

Some limitations of this study include differences between the two cohorts that may influence their forebrain projections, including gender mix and pre-injection history. Some evidence suggests that female gender affects mPFC (Knouse et al., 2022), although we have not found differences in previous work comparing MEMRI data between genders (Uselman et al., 2020). Lack of difference between sexes could be due to a lower sensitivity to such gender-dependent effects in our MEMRI methodology. Plasticity of medial forebrain projections has been hypothesized to play a role in drug use (Volkow et al., 1996; Frankin TR et al., 2002; Du et al., 2018) and in post -traumatic stress and anxiety disorders (Vertes, 2006; Sotres-Bayon and Quirk, 2010; Kesner and Churchwell, 2011; Laine et al., 2017; Padilla-Coreano et al., 2019). Thus, evidence exists that this circuit or network from mPFC into the limbic system may change in response to experience. It will be of interest to measure functional connections predicted here by more standard electrophysiological approaches. This study relied on a specific method for labeling and tracking neural projections. Therefore, it is possible that our method may miss some projections or labeled some projections incorrectly. Additional experiments using an alternative methodology may be needed to validate our observations. A next experiment would be to expose mice to cocaine and determine effects on these two projection fields in addicted *versus* naïve animals, possibly with MEMRI or other alternative mapping technology. It would be of interest if one or the other or both projection fields were redirected to alternate distal targets. Another experiment will be to test how early life adversity (ELA) affects mPFC projection anatomy in adults, which would inform on developmental influences on mPFC control implicated in the known vulnerability after ELA to drug abuse and anxiety disorders.

Here, Mn(II) distal accumulation serves as a proxy for axonal transport out axons to their distal, pre-synaptic termini and subsequent post-synaptic target, as we have done for hippocampal projections (Medina et al., 2019). We assume that this tracing is anterograde, occurs at similar rates between groups, and is primarily functional, i.e., afferent projections that are electrically active. Mn(II) transport continues within axons in the absence of electrical activity. We previously found that Mn(II) does not accumulate in the superior colliculus in blind mice after injection in the eye, despite entering intact retinal ganglion cells and traveling down their axons in the optic nerve (Bearer et al., 2007a). Thus, post-synaptic accumulations depend on active synapses. In sighted mice, Mn(II) crosses synapses from retinal ganglion cells into the superior colliculus, accumulating in post-synaptic neurons. Retrograde transport of Mn(II) has also been reported (Matsuda et al., 2010), in which case our data would detect both afferent and efferent connections between mPFC and deeper brain regions, although retrograde trans-synaptic tracing is unlikely in that scenario. While the injection of MnCl_2_ at this concentration and volume appears safe in mouse, rat, or even non-human primates (Uselman et al., 2022), there may be an acute excitatory event in neurons occasioned by a brief, locally high concentration of cation. This effect could drive the Mn(II) across synapses not normally active at the time of tracing. Our results do not distinguish between direct and indirect, i.e., trans-synaptic and polysynaptic circuits. We can only speculate that delayed arrival at locations fairly proximal to the injection site, such as the RSP and HPF, is occasioned by the necessity of crossing multiple synapses. Thus, the early, at 6 h, intensity increase in thalamus or striatum could, in principle, be because these locations are way-stations that accumulate tracer and then direct it onwards to other distal locations. Finally, our results support and extend reports on ACA projections revealed with viral tracers (Ma et al., 2022; Shi et al., 2022), albeit with some differences possibly due to location and volumes of injectate or to the underlying biology of the endogenous transport system and its interaction with the tracer.

In conclusion, this study mapped brain-wide projections from the ACA and IL/PL in living mice by MEMRI, quantified distal accumulations and compared them statistically. Significant differences were identified in key regions implicated in emotional states and their regulation, such as nuclei of the amygdala, hypothalamic subdomains, and periaqueductal gray, as well as in monoaminergic systems for serotonin and norepinephrine. Further experiments at the cellular level will be needed to learn more about rates of transport within these projections and the rate Mn(II) crosses synapses, as well as at the electrophysiological level to test the responses of predicted downstream targets to localized mPFC activities. Next experiments with MEMRI should focus on the plasticity of these projections in response to experiences and/or drug use.

Finally, the imaging tools gained from this work can be reapplied towards other questions concerning how specific cellular activities contribute to the wellbeing of the intact living organism, even in systems such as lung, heart, liver, thereby extending cell biological knowledge to encompass the living organism. Almost 30 years after George Palade, Albert Claude and Christian de Duve received the Nobel Prize for their discoveries on the structural and functional organization of the cell using imaging of fixed tissue combined with biochemistry of isolated organelles (1974), Paul Lauterbur and Sir Peter Mansfield received the Nobel for imaging living animals in 4D by MRI (2003). It is time we cross dimensional boundaries and apply MRI to study cellular physiology within the whole living organism.

## Data Availability

The raw data supporting the conclusion of this article will be made available by the authors, without undue reservation.
