# Supplementary material for: Harnessing axonal transport to map reward circuitry: Differing brain-wide projections from medial prefrontal cortical domains
Source: Front Cell Dev Biol. 2023 Nov 30;11:1278831. doi: 10.3389/fcell.2023.1278831 (PMC10720719; doi:10.3389/fcell.2023.1278831)
Supplement: Supplementary file 1 [file DataSheet1.pdf]

Supplementary Material

Harnessing axonal transport to map reward circuitry: Differing brain-wide projections from medial prefrontal cortical domains

E. L. Bearer, C. S. Medina, T. W. Uselman, R. E. Jacobs

Correspondence:
Elaine L. Bearer
ebearer@salud.unm.edu; ebearer@caltech.edu; elaine.bearer@gmail.com

1 Supplementary Data List

Table S1. Abbreviations (pdf)
Table S2. ROI coordinates (pdf
Table S3. ROI Measurements (excel)
Table S4. ROI Statistics (pdf)

Supplementary Fig. S1. Behavior
Supplementary Fig. S2. Injection site overlays
Supplementary Fig. S3. Coronal slices of SPM maps after ACA injection.

Supplementary Table S1. Abbreviations used in the column graphs in Fig. 6 and 8, in the order in which the columns appear in the graph. Nomenclature is according to the Allen Institute for Brain Science Mouse Brain Reference Atlas.

Abbreviation	Nominal Label	Abbreviation	Nominal Label
AAA	Anterior amygdalar area	MO	Somatomotor areas
ACA	Anterior cingulate area	MOBgl	Main olfactory bulb glomerular
ACB	Nucleus accumbens (a.k., NAc)	MOBgr	Main olfactory bulb granule layer
aco	Anterior commissure olfactory limb	MOBipl	Main olfactory bulb inner plexiform layer
AM	Anteromedial nucleus of thalamus	MOBmi	Main olfactory bulb mitral layer
AOB	Accessory olfactory bulb	MOBopl	Main olfactory bulb outer plexiform layer
AON	Anterior olfactory nucleus	MS	Medial septal nucleus
aot	Accessory optic tract	MY	Medulla
AV	Anteroventral nucleus of thalamus	NDB	Diagonal band nucleus
BLA	Basolateral amygdala nucleus	NOD	Nodulus X
BST	Bed nuclei of the stria terminalis	onl	Nerve layer of main olfactory bulb
CA1-CA3	Field ca1 ca2 ca3 pyramidal layer	opt	Optic tract
CB	Cerebellum	ORB	Orbital area
cc	Corpus callosum	OT	Olfactory tubercle
CEA	Central amygdalar nucleus	P	Pons
CLI	Central linear nucleus raphe	PA	Posterior amygdalar nucleus
CM	Central medial nucleus of the thalamus	PAG	Periaqueductal gray
COA	Cortical amygdala area	PB	Parabrachial nucleus
CP	Caudoputamen	PCG	Pontine central gray
CS	Superior central raphe nucleus	PF	Parafascicular nucleus
CTX	Cerebral cortex	PG	Pontine gray
DEC	Declive VI	PL	Prelimbic area
DG	Dentate gyrus	PO	Posterior complex of the thalamus
DP	Dorsal peduncular area	PRN	Pontine reticular nucleus
DR	Dorsal raphe nucleus	PT	Parataenial nucleus
em	External medullary lamina	PTL	Posterior parietal association areas
EPd	Endopiriform nucleus dorsal part	PVT	Paraventricular nucleus of the thalamus
fi	Fimbria	PYR	Pyramus VIII
FOTU	Folium-tuber Vermis VII	RE	Nucleus of reunions
FS	Fundus of striatum	RN	Red nucleus
GPe	Globus pallidus	RSP	Retrosplenial area
GR	Gracile nucleus	RT	Reticular nucleus of the thalamus
HPF	Hippocampal formation	SEZ	Subependymal zone
HY	Hypothalamus	SI	Substantia innominata
ILA	Infralimbic area	SIM	Simple lobule
IMD	Intermedial dorsal thalamus	sm	Stria medullaris
int	Internal capsule	SNc	Substantia nigra compact part
IPN	Interpeduncular nucleus	SNr	Substantia nigra reticular part
LA	Lateral amygdala nucleus	SPA	Subparafascicular area
LC	Loc coeruleus	SS	Somatosensory areas
LGd	Lateral geniculate complex dorsal part	st	Stria terminalis
lot	Lateral olfactory tract body	TT	Taenia tecta dorsal part
LP	Lateral posterior nucleus of the thalamus	UVU	Uvula IX
LSc	Lateral septal nucleus caudal part	V3	Third ventricle
LSr	Lateral septal nucleus rostral part	VAL	Ventral anterior lateral thalamic complex
MB	Midbrain	vhc	Ventral hippocampal commissure
MD	Mediodorsal nucleus of thalamus	VL	Lateral ventricle
MEA	Medial amygdalar area	VM	Ventral posterolateral thalamus
MEP	Median preoptic nucleus	VPL	Ventral posteromedial nucleus of the thalamus
MG	Medial geniculate complex	VPM	Ventral medial thalamic nucleus
MH	Medial habenula	VTA	Ventral tegmental area
MM	Medial mammillary nucleus	ZI	Zona incerta

Supplementary Table S2. Coordinates for Regions of Interest Analysis relative to Bregma (in mm)

**Region of Interest**	**Hemisphere**	**Coordinates**		
		**ML**	**DV**	**AP**
DS	Left	-1.5	0.8	2.7
	Right	1.4	0.8	3.7
GP	Left	-1.6	-0.3	4.7
	Right	1.7	-0.3	4.7
RNT	Left	-1.2	-0.5	4.2
	Right	1.3	-1.0	4.1
NAc/ACB	Left	-1.0	1.0	5.5
	Right	1.2	1.0	5.4
BLA	Left	-3.1	-2.0	5.9
	Right	3.6	-2.0	5.6
SNr	Left	-1.3	-3.1	5.4
	Right	1.5	-3.0	5.4
VTA	Left	-0.8	-3.0	5.2
	Right	0.7	-3.0	5.1
LC	Left	-0.9	-5.7	4.4
	Right	0.8	-5.7	4.6

Bregma locations corresponding to FSL (*fslroi*) defined 3 x 3 x 3 voxel cubes.

Supplementary Table S3. ROI Measurements

See Supplementary Table S3, a separate file available as Excel.

Supplementary Table S4. ROI Statistics (pdf)

Supplementary Table S4. Statistics for ROI within and between group analyses Figures 5 and 7 in the Main text.

Statistics for ACA within group analysis for differences at 6h vs 24h

**ROI**	**t.ratio**	**p value**	**Asterisks**
DS-L2	3.289	0.0006	*** <0.001
GP_L	-2.861	0.0045	** <0.01
GP_R	-4.304	<0.0001	****
NAC_R2	-1.682	0.0936	* <0.1
RNT_L2	3.212	0.0014	* <0.01
RNT_R2	4.468	<0.0001	****
SNR_L	-2.788	0.0056	** <0.01
SNR_R	-5.578	<0.0001	****
LC_L	3.124	0.0019	** <0.01
LC_R	2.680	0.0077	** <0.01

Statistics for between ACA and IL/PL cohorts at 24h post-injection

Region	t.ratio	p value	Asterisks
DS_L2	0.39	0.0642	* <0.1
DS_R2	3.917	0.0009	**** <0.001
RNT_L2	2.1	0.0486	** <0.05
BLA_L	-1.692	0.106	+ <= 0.2
SNr_R	3.624	0.0017	***<0.005

2.2 Supplementary Figures

Supplementary Figure S1: Time spent not moving

Mice were video recorded during the last 10m of 30m time spent in a custom arena at two timepoints before the forebrain injections: At baseline before any handling and at 23 days after handling, imaging and housing. Time spent not-moving within each 1-minute interval was tabulated in Ethovision and results graphed in Excel (Microsoft Office). Statistical comparisons were performed in R by ANOVA between these two time points. A small but statistically significant difference was found between baseline and 23d ($p < 0.01$).

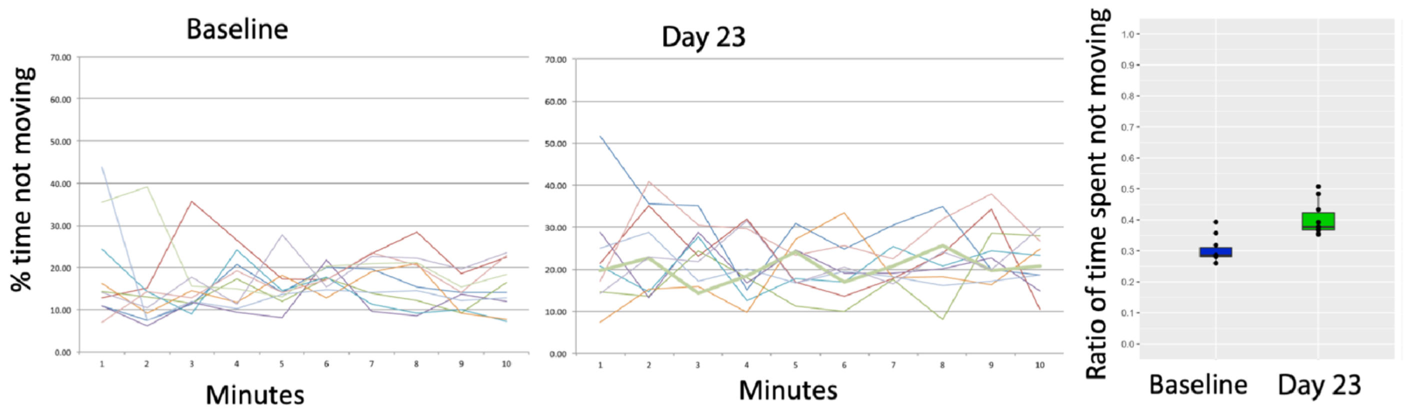

Supplementary Figure S2. Statistical maps of ACA and IL/PL injection sites

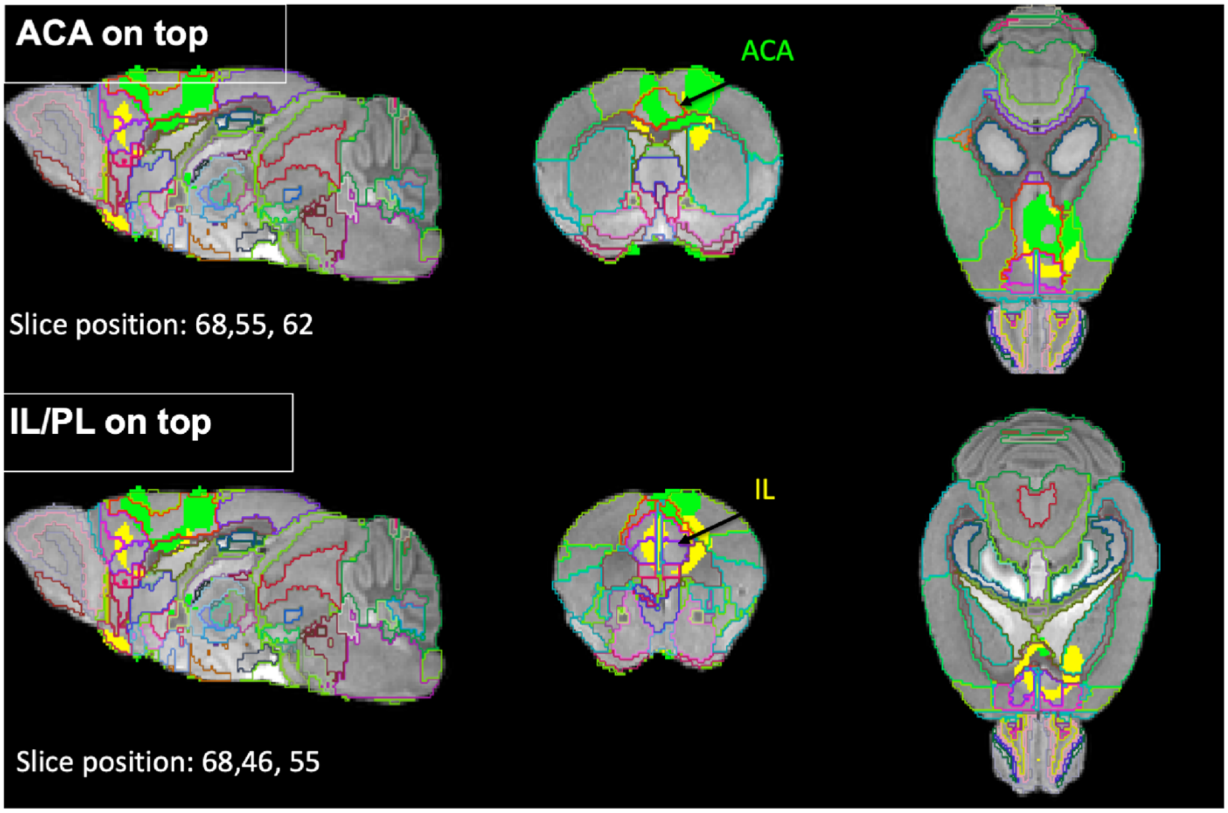

The 30m post-injection images for each cohort were compared to pre-injection image by a within-group paired t-test in SPM. Resultant maps of significantly enhanced voxels at a threshold of $p < 0.05$ FDR corrected (T values: IL/PL, T = 4.75; ACA, T = 4.05) were overlaid on the template image and with *InVivo Atlas* v.10 on the last layer to define position. Slice positions are indicated as voxel positions in the 3D dataset. Note that slice position of the injection halo representing Mn(II) diffusion out of the injection site differs in AP dimension by 9 voxels (0.9mm) and in the DV dimension by 7 voxels (0.7mm), and that there is some overlap in the region between the two sites. Bright pink outline delineates the ACA, and purple the Infralimbic segments as indicated on the coronal slices.

Supplementary Figure S3. Coronal slices from SPM overlays after ACA injection.

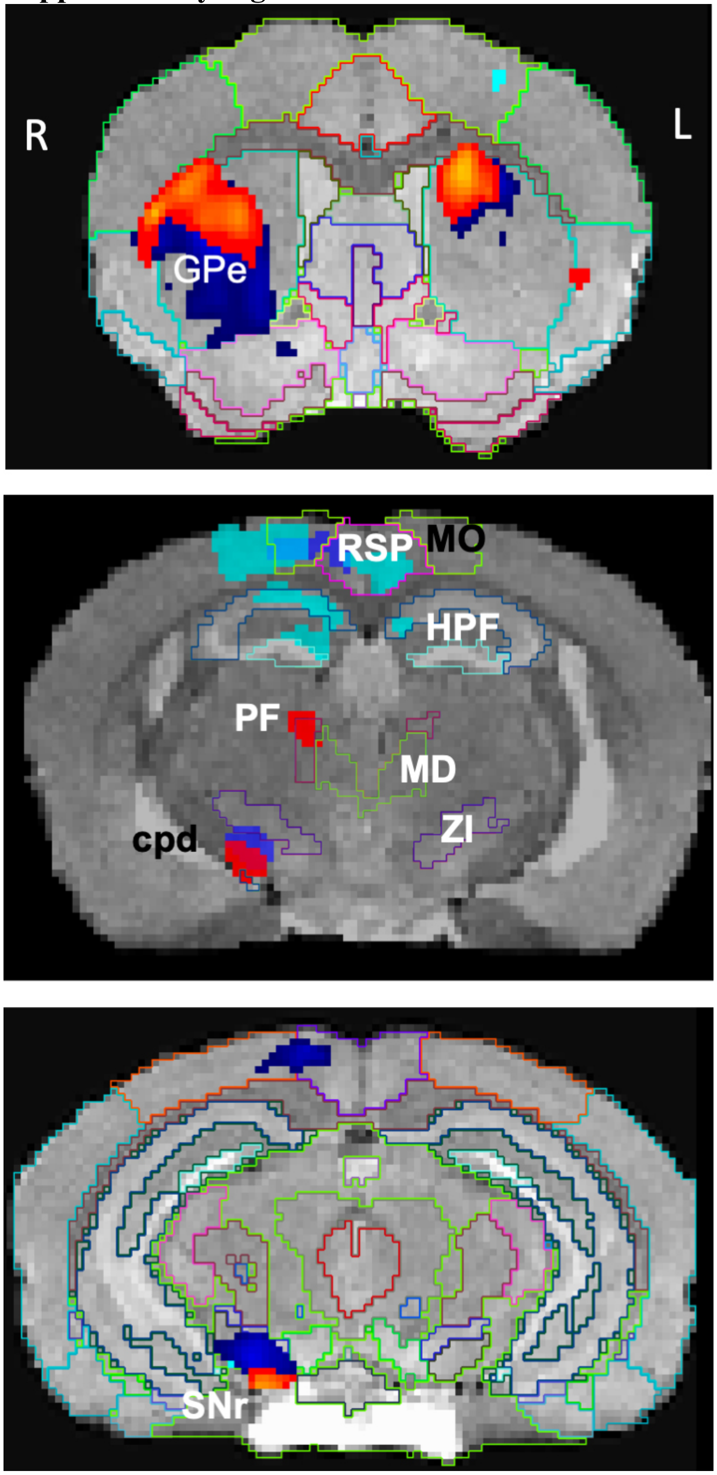

Shown are three coronal slices showing regions with statistically significant accumulations (T = 5.56, p<0.01 FDR) for all projections. Color coding is the same as for Figure 6 in the main text, red, 6h>30m; blue, 24h>6h; and turquoise, 24h > 6h. Slice positions shown in the corner of each image.

Top panel shows accumulation in the Globus pallidus (GPe) that progresses from 6h (blue) to 24h (red), with greater accumulation on the injection side (R). Mouse is facing forwards.

Middle panel shows statistically significant intensity increases due to Mn(II) accumulations differ between time points. Significantly, the Parafascicular nucleus of the thalamus accumulates signal first, appearing in the 6h>30m (red) map (Parafascicular nucleus, PF). Note that the Mediodorsal thalamic nucleus (MD), a known direct projection from the motor cortex (MO) is not highlighted at any time point. Signal also appears in this 6h map in a region running below the Zona incerta (ZI, purple outline). The ZI itself, another direct target of MO, has no signal at any time point. In scrolling through slices, signal in the cpd can be followed into the substantia nigra reticulata (SNr), where signal is robust at all timepoints. This trajectory below the ZI may represent the cerebral peduncle (cpd), which we purposefully had omitted from our segmentation analysis as cpd is a fiber bundle as we expected ACA projections would be diffuse and not appear in fiber bundles. However, others have recently reported transport in cerebral peduncle after ACA injection of a viral histologic tracer (Shi et al 2021).. At 24h (dark blue), signal in the cpd continues to be present and also newly appears in the retrosplenial area (RSP). The lack of signal in the cortex at 6h (no red) suggests that signal in the retrospenial area is not from Mn(II) diffusion out of the injection site, but rather transport from some other area, either through direct or trans-synaptic connections.

Signal appears only in comparisons between 6h and 24h (turquoise) in some regions, which may indicate slower arrival of the Mn(II), even in those that are closer as the crow flies to the injection site than the SNr. These late appearing accumulations appear in hippocampus (CA1 and part of the dentate on the injected side (R), and some in the CA1 field on the contralateral (L) side,) as well as in retrosplenial (RSP) and motor cortex (MO).

Lower panel shows statistically significant accumulation in substantia nigra reticulata (SNr) at 6hr (red) and 24h (dark blue). In cortex in this same slice, it can also be seen that signal arrives at the cortex at 24h.
